# Infection assessment tools for acute and chronic wounds: a scoping review

**DOI:** 10.1590/1980-220X-REEUSP-2024-0392en

**Published:** 2025-05-02

**Authors:** Daniella Cristina Julio Lima, Graciele Oroski Paes

**Affiliations:** 1 Universidade Federal do Rio de Janeiro, Escola de Enfermagem Anna Nery, Departamento de Enfermagem Fundamental, Rio de Janeiro, RJ, Brazil.

**Keywords:** Wound Healing, Risk Assessment, Wound Infection, Diagnostic Techniques and Procedures, Nursing

## Abstract

**Objective:**

To map the tools available in the literature for assessing wound infection.

**Method:**

This is a scoping review conducted from the *Joana Briggs Institute Manual for Evidence Synthesis*, in the databases of the Virtual Health Library, PubMed, Scopus, *Cumulative Index to Nursing and Allied Health Literature* and the academic literature repository, Google Scholar. The research question was developed based on the acronym PCC (Population/Concept/Context), resulting in the following formulation: “Which tools available in the literature assess wound infections?”

**Results:**

The analysis included 32 wound infection assessment tools. Of these, 26 using clinical signs and symptoms of infection distributed across scales, checklists, classification systems, and completion forms were identified. In addition to these, 5 electronic devices and 01 *software* were identified.

**Conclusion:**

The diversity of instruments highlights the complexity of wound infection management, emphasizing the need to discuss their applicability, benefits, and limitations to select the best evidence-based instrument.

## INTRODUCTION

The treatment of infected wounds represents a challenge for health professionals due to the complexity involved in their management. This process includes understanding the biopsychosocial and environmental factors that interfere in the care of individuals with acute and chronic wounds. In addition, broad knowledge of the various factors influencing the healing process is required, as well as of the treatment options available, which vary according to accessibility and availability of resources and periodic monitoring of these patients^(1, 2, 3)^.

The presence of a wound, especially chronic ones such as pressure ulcers, neuropathic, diabetic and vascular ulcers, negatively impacts patients’ quality of life. These injuries often cause pain, limitations and social isolation, interfering with daily life activities^(3)^. They can develop into serious complications, such as osteomyelitis and sepsis, and can even lead to death. Furthermore, they entail high costs for the healthcare system due to prolonged treatments and hospitalizations in some cases^(1, 2)^.

Despite existing recommendations and guidelines for the treatment and management of wounds of different etiologies, there is no consensus in the literature on the best practice to be systematically and standardly applied in care. Many professionals still deal with variability in the application of best evidence and face difficulties in identifying and assessing the severity of infections consistently^(4, 5)^. In clinical practice, the management of wound infections is largely based on health professinals’ experiences and knowledge, often without specialization in the area of wound care^(4, 6)^.

Infection identification traditionally occurs through clinical and visual observation of wounds, without systematization and standardization in the evaluation. Diagnosing infection is complex, as the characteristics of the wound vary according to the etiology and the patient’s morbidities, which hinders the identification of the infectious process in some cases, especially in chronic wounds, which may present subtly or even be asymptomatic^(6, 7)^. The lack of structured tools to assist in this identification and decision-making results in an approach that is often subjective and dependent on the experience of the healthcare professional^(8, 9)^.

In the literature, there are few epidemiological studies on the prevalence and incidence of infected wounds, especially in Brazil. Most existing studies in the Brazilian context indicate specific results, generally restricted to institutional levels, without a specific monitoring system integrated into health networks, and present high variability, depending on the region of the country, the type of study, and the etiology of the wound analyzed(^7^).

The use of tools can be a strategy adopted in units that treat wounds as a way of standardizing their management and treatment, allowing for a more objective and systematic evaluation. This helps and guides the professional in the assessment in a structured manner, using validated and reliable instruments, especially with regard to the identification and assessment of wound infections^(7, 8)^. It is known that the inappropriate and indiscriminate use of antimicrobials can lead to microbial resistance. Therefore, the use of tools for these conditions allows early identification of infections and timely treatment^(9)^.

Therefore, accurate and effective assessment to identify wound infection is essential to ensure adequate treatment and prevention of additional complications. As explained, the use of tools for assessing wound infections serves as a subsidy to assist health professionals in the adequate identification of the infection, in decision-making for timely and safe treatment, in the effective and efficient management of the injury, as well as in a systematic and organized assessment^(7, 10, 11)^.

Between April and May 2024, a preliminary literature search was carried out, which did not identify scoping or systematic reviews aimed at mapping tools for assessing wound infection. No records of ongoing studies were found on the PROSPERO, *Open Science Framework* (OSF), or *Figshare* platforms either. The absence of studies providing a comprehensive and systematic view of the available tools highlights a significant gap in the literature and warrants this study.

This gap in the literature, combined with the significant impact of wound infections on individuals and healthcare systems – such as longer hospital stays, increased treatment costs, risk of serious complications and mortality^(1)^ –, highlights the clinical and healthcare relevance of the topic. This unprecedented and essential study aims to map the tools available in the literature for evaluating wound infections.

## METHOD

### Design of Study

This is a scoping review conducted based on the *Joana Briggs Institute Manual for Evidence Synthesis* ^(12)^ and in the reporting recommendations of the *checklist Prisma Extension for Scoping Reviews* (PRISMA – ScR)^(13)^, to ensure methodological rigor, clarity, and organization. The protocol for this review was registered with the OSF, under DOI 10.17605/OSF.IO/57C2E. The study was developed according to the following steps: elaboration of the research question, survey of relevant studies, selection, data extraction, and presentation of results.

### Research Question

The formulation of the research question was based on the acronym PCC (Population/Concept/Context), with the population (P) being “individuals with wounds”, the concept (C) “wound infection assessment tools”, and the context (C) “infected wounds”. With this mnemonic combination, the following guiding question was defined: Which tools available in the literature assess wound infections?

### Eligibility Criteria

The inclusion criteria for this scoping review encompassed all available full-text manuscripts that answered the research question and were aligned with the study objective. To expand the search results, there were no restrictions regarding methodological design, publication period, or language. The context of this review was broad, with no restrictions on the care setting or any specific area of knowledge. Articles published in journals and gray literature publications, such as course completion papers, theses and dissertations, were considered.

Any constructs, instruments, and technologies aiming at assessing wound infection were adopted as infection assessment tools for this study. Regarding etiology, both acute (such as burns, surgical and cut-contusion wounds) and chronic (such as pressure injuries, vascular, neuropathic and diabetic ulcers) injuries with healing by secondary intention were considered. Tumor wounds were excluded from the evaluation, as they have a specific pathogenesis that is different from other lesions.

### Search Strategy

The search strategy was built based on the Health Sciences Descriptors (DeCS) and Medical Subject Headings (MeSH), using the Boolean operators AND and OR to optimize the retrieval of relevant information. The combinations were developed in conjunction with a librarian, considering the specifications of each database, to cover the maximum possible range of topics of interest, thus ensuring robust and comprehensive data collection, as seen in Chart 1.

**Chart 1:** Search strategy in the databases - Rio de Janeiro, RJ, Brazil, 2024.

Search Strategy	Databases/repository	Articles found
(cicatrização) OR (cicatrização de feridas) OR (cicatrização de ferimentos) OR (wound healing) AND (estudo de avaliação) OR (estudos de avaliação) OR (evaluation study) OR (evaluation studies) OR (avaliação em enfermagem) OR (protocolos de enfermagem) OR (nursing assessment) OR (nursing protocols) AND (tool) OR (instruments) OR (ferramentas) OR (instrumentos) AND (infecção dos ferimentos) OR (infecção das feridas) OR (infecção da ferida) OR (infecção de feridas) OR (wound infection)	VHL	89
wound healing AND wound infection AND tool	CINAHL	112
wound healing AND evaluation study OR evaluation studies OR nursing assessment OR nursing protocols AND wound infection AND tool	PubMed	193
wound healing AND wound infection AND tool AND nursing	Scopus	40
“wound infection” AND “wound healing” AND “consensus document” OR “tools” AND “clinical practice”	Google Scholar	6.720

The selected databases included the *Cumulative Index to Nursing and Allied Health Literature* (CINAHL), Scopus, the Virtual Health Library (VHS,) and PubMed, due to their ability to bring works from different areas of health and nursing together, providing a wide variety of indexes. This choice ensured comprehensive and diverse coverage of the available literature. Additionally, gray literature was included, as recommended by JBI, through Google Scholar. The searches took place between May 1 and 17, 2024.

### Study Selection

After searching the databases, the results found were transferred to the software Mendeley, where the identification and removal of duplicates were carried out. After removing duplicate works, articles were selected through reading of titles and abstracts, based on the criteria previously established in the study. The pre-selected papers were read in full to check for their permanence and relevance.

The information from the documents selected for analysis was independently extracted by two reviewers, using spreadsheets from Microsoft Excel®. A third reviewer participated in the validation of the information and in the discussion to establish consensus among the authors, when required. The manuscript selection process flow is shown in diagram form, as per PRISMA-ScR recommendations.

### Data Extraction

Data extraction was defined and adapted according to the JBI manual to select the following relevant information: year of publication, source of extraction of the tool or title of the manuscript, since some works were extracted from the same source, type of infection assessment tool, wound etiology, and the main characteristics of infection assessed by the tools.

### Data Presentation

The extracted data were presented in table form, based on the categorized data. A narrative presentation of the information was carried out, considering the objective of this scoping review. To highlight the most common clinical characteristics identified in the data found, a word cloud was created using the *Word Art website*. This visual resource facilitates the understanding of findings that appear most frequently in studies.

### Ethical Aspects

This scoping review was constructed exclusively based on publicly available secondary literature, without involving primary data or information identifying individuals. Therefore, in accordance with current ethical guidelines, there was no need to submit to the Research Ethics Committee, nor to request a Free and Informed Consent Form.

## RESULTS

According to the previously established search strategies, the research was conducted in the selected databases and academic repositories, resulting in the following findings: 89 articles in VHL, 112 in CINAHL, 193 in PubMed, 40 in Scopus and 6,720 in Google Scholar, totaling 7,154 manuscripts. Then, duplicates were excluded using the software Mendeley, which resulted in the removal of 44 documents, leaving a total of 7,110 articles for the reading of titles and abstracts.

**Figure 1:**
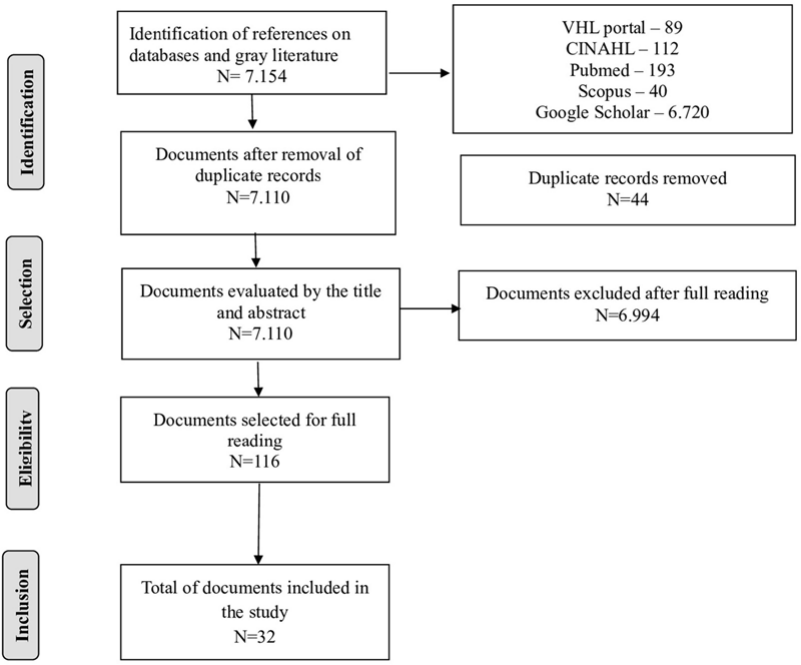
PRISMA-ScR flow diagram of the review publication selection process. Rio de Janeiro, RJ, Brazil, 2024.

After reading and analyzing the titles and abstracts, 116 manuscripts were selected for full reading, based on the eligibility criteria and the research question. Of these, 32 articles were finally included in the analysis and discussion, as described in the search and selection process flowchart presented in Figure 1.

After analyzing the selected articles, Chart 2 was created, compiling the main information found in the selected manuscripts, to facilitate the clear and concise presentation of the data. The chart was organized and separated by year of publication, source or title of the manuscript, type of tool found, name of the tool, wound etiology, infection characteristics assessed by the tool and country(ies) of the studies.

**Chart 2:** Characterization of the publications included in the review – Rio de Janeiro, RJ, Brazil, 2024.

	Year/Source or title of the manuscript	Tool type	Tool name	Wound etiology	Infection characteristics assessed by the tool	Country (ies) of study
1	2023/Wound infection in clinical practice Principles of best practice do International Wound Infection Institute^(9)^	Classification System	IDSA System	Diabetic ulcer	Clinical signs and symptoms such as: edema, erythema, purulent exudate, pain, heat, in addition to hemodynamic changes.	Netherlands, Australia, and Portugal
2	2023/Nursing care for people with diabetic foot wounds: construction of an instrument^(14)^	Filling form	Instrument for managing nursing care for people with diabetic foot	Diabetic ulcer	Clinical signs and symptoms such as: purulent exudate, pain, erythema, edema, foul odor, heat, in addition to hemodynamic changes.	Brazil
3	2023/Validation of a Wound Tool for Assessment of Surgical Wounds in Infants^(15)^	Classification system	University of Alberta Surgical Wound Assessment Tool	Surgical wound	Clinical signs and symptoms such as: purulent exudate, presence of exudate and changes in the skin around the wound (unspecific changes), necrosis or presence of a visible prosthesis.	Canada
4	2022/Development of a surgical wound assessment tool to measure healing and risk factors for delayed wound healing in Vietnam: a Delphi process^(16)^	Wound assessment construct	SWAT	Surgical wound	Clinical signs and symptoms such as: edema, erythema, exudate, odor, pain, among other signs.	Vietnam
5	2022/Instrument for the evaluation of the chronic wounded patient: clinical, care and financial indicators^(17)^	Filling form	Instrument for assessing patients with chronic wounds at Universidade Federal de Minas Gerais	Chronic wounds of various etiologies	Clinical signs and symptoms such as: detachment, necrosis, exudate, edema, hardening, odor, color of the skin around the wound (not specified); signs of critical colonization in the wound bed and signs of infection in the wound bed (the latter without specification).	Brazil
6	2022/Wound Infection in Clinical Practice -Principles of best practice^(9)^	Conceptual model	Wound Infection Continuum – International Wound Infection Institute (WIC – IWII)	Acute and chronic wounds of various etiologies	Clinical signs and symptoms such as: hypergranulation; friable granulation; increased exudate, delayed healing, erythema, heat, edema, purulent exudate, wound enlargement, pain, odor, in addition to other signs and symptoms and hemodynamic changes.	USA
7	2021/Post-dishcarge surveillance in surgical site infection: validation of an instrument^(18)^	Post-discharge surgical site surveillance form	Post-discharge surveillance for detection of surgical site infection at Universidade de São Paulo	Surgical wound	Clinical signs and symptoms such as: purulent exudate, edema, pain, heat, erythema, dehiscence, among other signs and symptoms besides hemodynamic changes.	Brazil
8	2021/Contribution of software for recording, monitoring, and evaluating wounds^(19)^	Software	Wounds Monitoring	Acute and chronic wounds of various etiologies	Clinical signs and symptoms such as: exudate, signs of inflammation and/or infection (does not specify which), among others.	Brazil
9	2020/Validity of DMIST for monitoring healing of diabetic foot ulcers^(20)^	Scale	DMIST	Diabetic ulcer	Clinical signs and symptoms such as: hardening, purulent exudate, unpleasant odor, among other signs and symptoms besides hemodynamic changes.	Japan
10	2020/Therapeutic Index for Local Infections score validity: a retrospective European analysis^(21)^	Classification system	Therapeutic Index for Local Infections score	Lower limb ulcers of various etiologies	Clinical signs and symptoms such as: erythema, heat, edema, pain, delayed healing, odor, purulent exudate, among other “non-direct indicators of infection”.	Germany
11	2020/Wound Infection in Clinical Practice - Principles of best practice^(9)^	Classification system	Infection Management Pathway	Acute and chronic wounds of various etiologies	Clinical signs and symptoms such as: erythema, edema, purulent exudate, pain, foul odor, delayed healing, increased erythema, heat, hypergranulation, friable granulation, wound enlargement, in addition to other signs and symptoms and hemodynamic changes.	Germany
12	2020/Validation of the Harikrishna Peripheral Skin Classification for wound assessment^(22)^	Classification system	Harikrishna Peripheral Skin Classification (HPSC)	Acute and chronic wounds of various etiologies	Classifies perilesional skin for infection, through signs such as: inflammation without infection, inflammation with infection, atypical – senescent cells/cancer/subcutaneous emphysema.	Malaysia
13	2020/Management tools in nursing care for children with pressure injury^(23)^	Fill-in form and a Flowchart	Instrument for systematizing nursing care for children with pressure injuries and Flowchart of risk and prevention of pressure injuries at Universidade Federal do Espírito Santo	Pressure ulcer	Clinical signs and symptoms such as: edema, increased temperature, hyperemia, and increased necrotic tissue.	Brazil
14	2019/Use of a bacterial fluorescence imaging device: wound measurement, bacterial detection and targeted debridement^(24)^	Device	MolecuLight	Diabetic, venous, arterial ulcers, surgical wounds and pressure ulcers	Assesses infection through bacterial load.	Canada
15	2019/Evaluation of Wound Photography for Remote Postoperative Assessment of Surgical Site Infections^(25)^	Photographic record through the use of *smartphones*	Not applicable	SSI (Surgical Site Infection) or SSO (Surgical Site Occurrence)	Signs of infection reported by patients as present or absent and use of photographic images, associated or not with the report of present or absent infection.	USA
16	2018/Importance of postprocedural Wound, Ischemia, and foot Infection (WIfI) restaging in predicting limb salvage^(26)^	Classification system	WIfI	Lower limb ulcers of various etiologies	Clinical signs and symptoms such as: local infection involving only the skin and subcutaneous tissue, local infection with erythema >2 cm or involving structures deeper than the skin and subcutaneous tissues and local infection with signs of systemic inflammatory response syndrome.	USA
17	2018/Diagnostic value of fluorine-18 deoxyglucose positron emission tomography/computed tomography in deep sternal wound infection^(27)^	Positron emission computed tomography (PET/CT)	Not applicable	Surgical wound	Signs of infection through the affected area shown in the image.	China
18	2018/Elaboration of an algorithm for wound evaluation and treatment^(28)^	Algorithm	Algorithm for wound assessment and treatment at Universidade do Vale do Sapucaí	Acute and chronic wounds of various etiologies	Clinical signs and symptoms such as: heat, erythema, edema, pain, purulent exudate, in addition to hemodynamic changes.	Brazil
19	2017/The inter-rater reliability between nurse-assessors clinically assessing infection of chronic wounds using the WUWHS criteria^(29)^	Filling form	WUWHS	Chronic wounds of various etiologies	Clinical signs and symptoms such as: pain, delayed healing, edema, friable granulation tissue, foul odor, discoloration, increased exudate, induration, erythema, in addition to hemodynamic changes.	Netherlands
20	2017/Wound Infection in Clinical Practice -Principles of best practice^(9)^	Classification system	WIRE	Acute and chronic wounds of various etiologies	Clinical signs and symptoms such as: pain, necrotic tissue, friable granulation, exposure of underlying organs, delayed healing, erythema, heat, edema, odor, exudate, among others.	United Kingdom
21	2017/Inter-rater and intra-rater reliability outcomes of a rapid bacteria counting system with pressure ulcer samples^(30)^	Dispositivo Panasonic Healthcare DU-AA01NP-H	Not applicable	Pressure ulcer	Assesses infection through bacterial load.	Japan
22	2016/The Wound Trend Scale: A Retrospective Review of Utility and Predictive Value in the Assessment and Documentation of Lower Leg Ulcers^(31)^	Scale	WTS	Lower limb ulcers of various etiologies	Clinical signs and symptoms such as: exudate, necrotic tissue and debridement, depth, purulence, periwound skin, edema, pain and risk of infection (erythema, heat, edema and pain, in addition to infection screening through exams).	Canada
23	2016/Evaluation of a Surgical Site Discharge Teaching Tool Using Pictures and a Mirror^(32)^	Health education for teaching self-assessment of surgical incisions.	Not applicable	Surgical wounds, specifically after laparotomy	Clinical signs and symptoms such as: heat, drainage, purulent exudate, erythema, edema, pain/sensitivity, itching, among other signs and symptoms.	USA
24	2015/Cultural adaptation and validation for the Portuguese population of a chronic wound monitoring instrument: RESVECH 2.0 scale^(33)^	Scale	RESVECH 2.0	Chronic wounds of various etiologies	Clinical signs and symptoms such as: pain; erythema, edema, heat, increased exudate, purulent exudate, friable tissue, stagnant wound, tissue compatible with biofilm, odor, hypergranulation, increased wound size, satellite lesions, tissue pallor, among other signs and symptoms.	Portugal
25	2015/Construction of a data collection instrument for people with wounds based on Wanda de Aguiar Horta’s theory^(34)^	Filling form	Data collection instrument for people with wounds based on the theory of Wanda de Aguiar Horta from the Universidade Federal da Paraíba	Diabetic ulcer	Clinical signs and symptoms such as: edema, heat, erythema, tissue increase, pain and exudate.	Brazil
26	2013/Reliability and validity of the Chinese version of DESIGN-R, an assessment instrument for pressure ulcers^(35)^	Filling form	DESIGN-R	Pressure ulcer	Clinical signs and symptoms, but does not specify which ones, as well as other general characteristics assessed of the wound such as: wound depth, exudate, size, infection, granulation and necrosis.	China
27	2012/A systematic review of the ASEPSIS scoring system used in non-cardiac-related surgery^(36)^	Classification system	ASEPSIS	Surgical wounds	Clinical signs and symptoms, but does not specify which ones, as well as other general characteristics assessed of the wound such as: serous secretion, erythema, purulent exudate, separation of deep tissues, isolation of bacteria, and hospitalization for more than 14 days.	United Kingdom
28	2006/Wound Infection in Clinical Practice - Principles of best practice^(9)^	Classification system	NERDS and STONES	Chronic wounds of various etiologies	Clinical signs and symptoms such as: non-healing wounds, oozing wounds, red and bleeding granulation tissue on the wound surface, debris on the wound surface, foul smell or odor from the wound, increase in size, increased temperature, probing or exposure of bone, new split areas or satellites injuries, exudate, erythema, edema.	Canada
29	2001/A tool to assess clinical signs and symptoms of localized infection in chronic wounds: development and reliability^(37)^	Checklist	Clinical Signs and Symptoms Checklist (CSSC)	Chronic wounds of various etiologies	Clinical signs and symptoms such as: increased pain in the ulcer area, erythema, edema, heat, purulent exudate, serous exudate associated with inflammation, delayed ulcer healing, discoloration of granulation tissue, friable granulation tissue, foul odor, satellite wounds, and pocketing at the ulcer base.	USA
30	1997/The Sessing Scale for measurement of pressure ulcer healing^(38)^	Scale	Sessing Scale	Pressure ulcer	Clinical signs and symptoms such as: purulent drainage, foul odor, necrotic tissue, in addition to septic symptoms.	USA
31	1997/Monitoring wound healing by odour^(39)^	Device with sensors	AromaScan	vascular ulcer	Aroma of lesions and its relationship with microorganisms present in the wound.	United Kingdom
32	1995/Wound registry: development and validation^(40)^	Form	State University of New York Completion Form	Traumatic wounds	Clinical signs and symptoms such as: Erythema, heat, tenderness or drainage, combined with clinical judgment, and short-term assessment of cosmetic appearance.	USA

**Figure 2:**
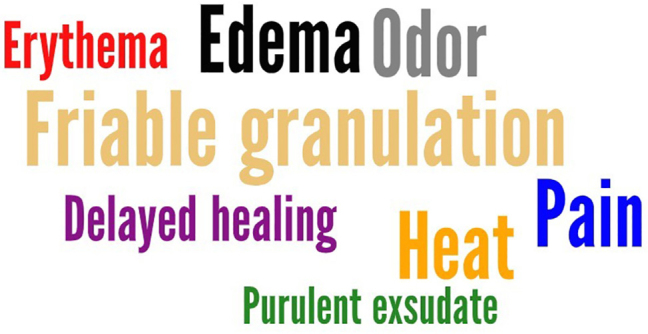
Word cloud of common signs and symptoms in publications. Rio de Janeiro, RJ, Brazil, 2024.

To help identify the main characteristics of infections in the studies analyzed, a word cloud was created, illustrated in Figure 2. This visual representation highlights the key clinical signs and symptoms of wound infections. The data presented reflect the most prevalent information found in the evaluated works.

The findings of this review identified 32 tools for assessing wound infection, with publications ranging from 1995 to 2023, with 13 works published in the last 5 years. Most of the tools found assess the infection qualitatively, through clinical signs and symptoms, corresponding to 26 instruments^(9, 14, 15, 16, 17, 18, 20, 21, 22, 23, 26, 28, 29, 31, 32, 33, 34, 35, 36, 37, 38, 40, 41, 42)^. The others evaluate using more sophisticated technological resources, such as electronic equipment and devices, totaling 5 tools, and only 1 uses software for assessment of injury and infection^(19, 24, 25, 27, 30, 39)^.

The tools assessing infection through the signs and symptoms presented by the wound are traditional constructs, which come in different forms, ranging from forms, scales, classification systems, checklists, acronyms, explanatory booklets and algorithms, which can be printed on paper to be filled in with a pen or on digital platforms. Such tools assess infection through direct observation of the skin, lesion bed, and perilesional skin. The signs and symptoms of infection assessed vary according to the specificities of each construct and the etiology of the wound assessed^(9, 14, 15, 16, 17, 18, 20, 21, 22, 23, 26, 28, 29, 31, 32, 33, 34, 35, 36, 37, 38, 40, 41, 42)^.

Instruments that assess wound infections using technological resources include equipment that uses positron emission imaging combined with computed tomography, quantitative assessment of microbial load with the use of immunofluorescence images, software for recording, monitoring and analyzing wounds (including signs and symptoms of infection), sensors for evaluating odor as a predictive indicator of infection, and also the use of smartphones to help identify wound complications such as infections^(19, 24, 25, 27, 30, 39)^.

Among the traditional tools that assess infection based on clinical signs and symptoms, the following are distinctive: *Clinical Signs and Symptoms Checklist* (CSSC), *Therapeutic Index for Local Infections* (TILI), *Infection Management Pathway, Wound Infection Continuum - International Wound Infection Institute* (WIC-IWII), *World Union of Wound Healing Societies* (WUWHS) and the acronyms ASEPSIS, WIRE, NERDS and STONES, WIfI, IWGDF/IDSA *System* ^(9, 21, 26, 29, 36, 37, 41, 42)^. Other instruments were also identified in the studies analyzed, although they do not have specific names^(14, 15, 16, 17, 18, 23, 28, 32, 34, 40)^. Classic signs and symptoms of infection mentioned in most of these tools include pain, heat, edema, erythema, purulent exudate, and odor^(9, 14, 15, 16, 17, 18, 20, 21, 22, 23, 26, 28, 29, 31, 32, 33, 34, 35, 36, 37, 38, 40, 41, 42)^.

Only the acronyms NERDS and STONES defined the number of signs and symptoms required to classify infection in a wound^(9)^. No other tool has specified the number of clinical features that must be present in the lesion for the diagnosis of infection^(9, 21, 26, 29, 36, 37, 41, 42)^. As traditional tools point out, no single sign or symptom reliably determines the presence or absence of infection in a wound. Therefore, the combination of multiple signs and symptoms indicative of infection should be used for a more accurate and safe clinical diagnosis^(9, 29)^.

The instruments also identified that some signs and symptoms are more specific and sensitive as predictors of infection than others. In addition, certain signs may manifest subtly or even not appear at all, depending on the type of wound and the patient’s clinical conditions^(9, 34, 37, 41)^. The work also demonstrated that signs of inflammation - heat, erythema, edema and pain – can be present in infected wounds, and are considered classic signs of infection^(9, 14, 15, 16, 19, 20, 23, 24, 27, 30, 31, 32, 33, 34, 31, 32, 33, 34, 36, 39, 40)^.

Other tools analyzed have, in their composition, an item or domain that evaluates infection, among other characteristics, although they were not specifically designed for this purpose. These tools include RESVECH 2.0, DESIGN-R, *Harikrishna Periwound Skin Classification* (HPSC), *Wound Trend Scale* (WTS), *Sessing Scale*, the DMIST and SWAT mnemonics, as well as other unnamed tools. The most frequently identified clinical signs and symptoms of infection include: purulent exudate, foul odor, erythema, edema^(16, 20, 22, 31, 33, 35, 38)^.

Regarding the tools that quantitatively assess the microbial load present in the lesion bed and its concentration, the device MolecuLight and the Panasonic appliance *Healthcare* DU-AA01NP-H are highlighted. These devices allow for less invasive and rapid bedside infection assessment^(24, 30)^. Additionally, the AromaScan equipment assists in monitoring wound progress and identifying possible infection based on the pattern of odors detected. Conductive polymer sensors, in turn, show changes in electrical resistance when exposed to a mixture of volatile chemicals(^39^).

The use of smartphones, also found in the mapping of tools for this research, can be used to aid in the assessment of surgical wound complications such as SSI (Surgical Site Infection) or SSO (Surgical Site Occurrence), including seroma, cellulitis, hematoma, dehiscence and abscess^(25)^. As for computed tomography, this technology has shown itself to be a possibility for identifying infection in deeper structures of the wound(^27^).

Regarding the geographical distribution of the tools, 18 were identified on the American continent, 7 in Brazil, 4 in Canada, and 7 in the United States^(9, 14, 15, 17, 18, 19, 23, 24, 25, 26, 31, 32, 34, 37, 38, 40)^. In Asia, 6 tools were found: 1 in Vietnam, 2 in Japan, 2 in China and 1 in Malaysia^(16, 20, 22, 30, 35)^. In Europe, 8 tools were located, distributed within Germany (2), the Netherlands (2), the United Kingdom (3), and Portugal (1)^(9, 21, 29, 33, 36)^. In other continents, no published works were found in this scoping review.

Among the ten traditional clinical tools, specific for evaluating infection through signs and symptoms, 6 are from the European continent^(9, 21, 29, 36)^ and 4 from the American continent^(9, 26, 37)^. The others present some item or domain for infection assessment, but are not specific for this purpose^(14, 15, 16, 17, 18, 19, 20, 22, 23, 28, 31, 32, 33, 34, 35, 38, 40)^. None of the Brazilian studies mapped in this review exclusively evaluate the infectious process in wounds^(14, 17, 18, 19, 23, 28, 34)^. 

## DISCUSSION

Mapping the evidence available in the literature reveals that wound infection assessment tools have different applicability, with a predominance of instruments based on traditional approaches, which assess infection through clinical signs and symptoms observed in the lesions. This finding is in line with the main international guidelines, which recommend analyzing wound characteristics to diagnose infection. This approach stands out for being practical, accessible, fast and viable for application in different clinical scenarios, in addition to presenting good validity and reliability, allowing early diagnosis^(9, 14, 15, 16, 17, 18, 20, 21, 22, 23, 26, 28, 29, 31, 32, 33, 34, 35, 36, 37, 38, 40, 41, 42)^.

Clinical signs play a fundamental role in monitoring the progression of infected wounds, being key elements in guiding therapeutic decisions and assessing the response to treatment^(8, 10, 29, 31, 33)^. Assessment tools allow these signals to be organized in a systematic way, promoting structured and more efficient monitoring^(7, 9, 11)^. Careful analysis of clinical signs helps healthcare professionals identify early changes in the condition of the lesion, enabling timely adjustments in therapeutic interventions and contributing to improved clinical outcomes^(8, 10)^.

Most tools that assess infection based on clinical signs and symptoms include the cardinal signs of inflammation: pain, heat, edema, and erythema. This is justified by the fact that the inflammatory process is present in infected wounds, making these signs also relevant indicators of infection. It is known that they are not specific for infection if evaluated in isolation, but they should be considered as predictors of infection in wounds that present these characteristics without an identified cause. According to some tools, signs and symptoms of infection are still classified into classic, subtle, and systemic signs^(8, 9, 14, 20, 21, 26, 29, 41, 42)^.

Technological tools for assessing wound infection are less frequent in the literature, possibly due to high cost and limited availability^(24, 27, 30, 39)^. However, these technologies can be particularly useful in contexts that require greater precision and agility. The reviewed studies suggest the need for further research to consolidate the use of these technologies in clinical practice. Despite this, they already demonstrate potential to offer a quick, safe, and less invasive diagnosis, favoring timely treatment and effective management of the infection^(9, 19, 24, 25, 27, 30, 39)^.

Regarding the types of wounds evaluated, it is observed that there is a diversity of etiologies, covering both acute and chronic wounds, which allows their application in different care contexts, such as primary, home, and hospital care^(9, 21, 26, 29, 36, 37, 41, 42)^. This finding is similar to Cardinelli et al.’s ^(11)^ results, who also identified assessment instruments applicable to different etiologies and clinical scenarios.

The literature presents a wide range of instruments for the evaluation of infected wounds, which vary from comprehensive tools capable of evaluating multiple lesions, to others more specific to certain etiologies^(9, 14, 15, 16, 17, 18, 20, 21, 22, 23, 26, 28, 29, 31, 32, 33, 34, 35, 36, 37, 38, 40, 41, 42)^. Knowing these instruments and their particularities is essential so that health professionals can adapt care in a more precise, safe, and effective way^(11)^.

The use of wound assessment tools has proven to be a technology capable of improving the quality of patient care, since it guides clinical practice in the treatment and management of these injuries based on the best evidence. Its use also allows the standardization of wound analysis, reducing subjectivity in the assessment. Although the literature points to these instruments as a possibility for improving the quality of care, there are few published studies proving the effectiveness of their routine and systematic use in clinical practice^(11, 43)^.

The application of these technologies in the management of patients with wounds can present significant benefits; therefore, a thorough evaluation is required for an appropriate selection, depending on the context in which the injuries are managed and the objective of the evaluation. Furthermore, it is essential to understand how to use these tools correctly, to avoid their inappropriate use and, consequently, erroneous assessments^(11, 43)^.

With regard to the assessment of infection, the consensus of *International Wound Infection Institute* (IWII), formulated in 2022, presents specific tools for identifying wound infections of different etiologies. Some of these are in the psychometric validation phase, while others have already been validated for clinical use. Additionally, they include analysis of risk factors for infection^(9)^.

In the Brazilian context, a scoping review mapped 51 instruments available in the literature for wound assessment, of which only eight were adapted and validated for use in Brazil. However, none of these instruments are specific for wound infections. Most of the tools translated into Portuguese focus on assessing the healing process, classifying injuries and adequately preparing the wound bed to optimize treatment^(11)^.

Besides the variety of instruments available, it is crucial to consider the validity and reliability of assessment tools. There is no tool considered the “gold standard” for identifying wound infections, as this choice depends on the type of injury, the evaluation scenario and the available resources. Furthermore, the healthcare team must be properly trained and qualified to use infection assessment tools safely and effectively. Regardless of the instrument chosen, it is known that a reliable and validated tool helps in the care of patients with wound infections, preventing complications and promoting better decision-making^(9, 11, 43)^.

Despite advances in science and technology applied to wound care, there is still a significant gap in the systematization of evaluation and validation of specific tools to identify wound infections. This scoping review seeks to contribute to filling this gap by mapping existing tools, providing a comprehensive overview of their characteristics. Moreover, the research highlights the lack of validated instruments in the Brazilian context for assessing infection.

This review results provide a basis for future clinical investigations and methodological studies aimed at creating and validating tools, strengthening evidence-based practice. By systematizing the data, this review seeks to assist health professionals in choosing appropriate methods to diagnose and manage wound infections, promoting the standardization of care and, consequently, better clinical outcomes for patients.

## CONCLUSION

The diversity of tools for assessing wound infection available in the literature reflects the complexity and multidimensionality of managing infected wounds. These instruments range from simple forms for manual completion and global assessment of the lesion, covering different aspects such as the presence of clinical signs and symptoms of infection, to the use of more advanced and technological equipment for analysis of microbial load and imaging exams.

The most commonly used assessment tools in clinical practice are based on clinical signs, and there are no validated instruments in Brazil to specifically identify wound infections. This study suggests the development of tools adapted to the Brazilian reality and the conduction of further research to validate the reliability, benefits, and limitations of these instruments in healthcare practice.
