# Supplementary material for: Infection assessment tools for acute and chronic wounds: a scoping review
Source: Rev Esc Enferm USP. 2025 May 2;59:e20240392. doi: 10.1590/1980-220X-REEUSP-2024-0392en (PMC12063538; doi:10.1590/1980-220X-REEUSP-2024-0392en)
Supplement: Translation

# Translation: Ferramentas de avaliação de infecção em feridas agudas e crônicas: revisão de escopo

**DOI:** 10.1590/1980-220X-REEUSP-2024-0392pt

**Authors:** Daniella Cristina Julio Lima, Graciele Oroski Paes

**Affiliations:** 1 Universidade Federal do Rio de Janeiro, Escola de Enfermagem Anna Nery, Departamento de Enfermagem Fundamental, Rio de Janeiro, RJ, Brasil.

**Keywords:** Cicatrização, Avaliação de risco, Infecção dos ferimentos, Técnicas e procedimentos diagnósticos, Enfermagem

## Abstract

**Objetivo:**

Mapear as ferramentas disponíveis na literatura para avaliação de infecção em feridas.

**Método:**

Trata-se de uma revisão de escopo conduzida a partir do *Joana Briggs Institute Manual for Evidence Synthesis*, nas bases de dados da Biblioteca Virtual em Saúde, PubMed, Scopus, *Cumulative Index to Nursing and Allied Health Literature* e do repositório de literatura acadêmica, Google Acadêmico. A pergunta de pesquisa foi elaborada com base no acrônimo PCC (População/Conceito/Contexto), resultando na seguinte formulação: “Quais ferramentas disponíveis na literatura avaliam infecção em feridas?”.

**Resultados:**

A análise incluiu 32 ferramentas de avaliação de infecção em feridas. Dessas, foram identificadas 26 que utilizam sinais e sintomas clínicos de infecção distribuídas entre escalas, listas de verificação, sistemas de classificação e formulários de preenchimento. Além dessas, foram identificados 5 dispositivos eletrônicos, e 01 *software*.

**Conclusão:**

A diversidade de instrumentos destaca a complexidade do manejo de infecção em feridas, enfatizando a necessidade de discutir sua aplicabilidade, benefícios e limitações para selecionar o melhor instrumento baseado em evidências.

## INTRODUÇÃO

O tratamento de feridas infectadas representa um desafio para os profissionais de saúde devido à complexidade envolvida em seu manejo. Esse processo inclui o entendimento dos fatores biopsicossociais e ambientais que interferem no cuidado de indivíduos com feridas agudas e crônicas. Além disso, é necessário um conhecimento amplo dos diversos fatores que influenciam o processo de cicatrização, as opções de tratamento disponíveis, que variam conforme a acessibilidade e a disponibilidade de recursos, e a necessidade de acompanhamento periódico desses pacientes^(1, 2, 3)^.

A presença de uma ferida, especialmente as crônicas, como lesões por pressão, úlceras neuropáticas, diabéticas e vasculares, impacta negativamente a qualidade de vida dos pacientes. Essas lesões costumam causar dor, limitações e isolamento social, interferindo nas atividades da vida diária^(3)^. Podem evoluir para complicações graves, como osteomielite e sepse, podendo, inclusive, evoluir para óbito. Além disso, acarretam altos custos para o sistema de saúde devido a tratamentos e hospitalizações prolongadas em alguns casos^(1, 2)^.

Apesar das recomendações e diretrizes existentes para o tratamento e gerenciamento de feridas de diversas etiologias, não há consenso na literatura sobre a melhor prática a ser aplicada de forma sistematizada e padronizada na assistência. Muitos profissionais ainda lidam com a variabilidade na aplicação das melhores evidências e enfrentam dificuldades em identificar e avaliar a gravidade das infecções de maneira consistente^(4, 5)^. Na prática clínica, o manejo das infecções em feridas é, em grande parte, baseado nas experiências e conhecimentos dos profissionais de saúde, muitas vezes sem especialização na área de tratamento de feridas^(4, 6)^.

A identificação da infecção ocorre tradicionalmente por meio da observação clínica e visual das feridas, sem uma sistematização e padronização na avaliação. O diagnóstico de infecção é complexo, pois as características da ferida variam de acordo com a etiologia e as morbidades do paciente, o que dificulta a identificação do processo infeccioso em alguns casos, principalmente nas feridas crônicas, que podem se apresentar de forma sutil ou até mesmo assintomática^(6, 7)^. A falta de ferramentas estruturadas que auxiliem nessa identificação e na tomada de decisão resulta em uma abordagem, muitas vezes, subjetiva e dependente da vivência do profissional de saúde^(8, 9)^.

Na literatura, há poucos estudos epidemiológicos sobre a prevalência e incidência de feridas infectadas, principalmente no Brasil. A maioria dos trabalhos existentes no contexto brasileiro aponta resultados pontuais, geralmente restritos a níveis institucionais, sem um sistema de monitoramento específico e integrado às redes de saúde, além de apresentar alta variabilidade, dependendo da região do país, do tipo de estudo e da etiologia da ferida analisada^(7)^.

O uso de ferramentas pode ser uma estratégia adotada nas unidades que tratam de feridas como forma de padronizar seu gerenciamento e tratamento, permitindo uma avaliação mais objetiva e sistematizada. Isso auxilia e direciona o profissional na avaliação de forma estruturada, por meio de instrumentos validados e confiáveis, principalmente no que tange à identificação e avaliação de infecções em feridas^(7, 8)^. Sabe-se que o uso inadequado e indiscriminado de antimicrobianos pode levar à resistência microbiana. Logo, o uso de ferramentas para essas condições permite a identificação precoce de infecções e o tratamento oportuno^(9)^.

Sendo assim, a avaliação precisa e eficaz para a identificação de infecção em feridas é fundamental para garantir o tratamento adequado e a prevenção de complicações adicionais. Conforme exposto, a utilização de ferramentas para a avaliação de infecção em feridas serve como um subsídio para auxiliar os profissionais de saúde na identificação adequada da infecção, na tomada de decisões para o tratamento oportuno e seguro, no gerenciamento eficaz e eficiente da lesão, bem como em uma avaliação sistematizada e organizada^(7, 10, 11)^.

Entre abril e maio de 2024, foi realizada uma pesquisa preliminar na literatura, que não identificou revisões de escopo ou sistemáticas voltadas ao mapeamento de ferramentas para avaliação de infecção em feridas. Também não foram encontrados registros de estudos em andamento nas plataformas PROSPERO, *Open Science Framework* (OSF) ou *Figshare*. A ausência de estudos que ofereçam uma visão abrangente e sistematizada sobre as ferramentas disponíveis evidencia uma lacuna significativa na literatura e justifica a realização desta pesquisa.

Essa lacuna na literatura, aliada ao impacto significativo das infecções em feridas nos indivíduos e nos sistemas de saúde – como maior tempo de hospitalização, aumento de custos com o tratamento, risco de complicações graves e mortalidade^(1, 2, 3)^ –, ressalta a relevância clínica e assistencial do tema. Este estudo, inédito e essencial, tem como objetivo mapear as ferramentas disponíveis na literatura para avaliação de infecção em feridas.

## MÉTODO

### Tipo de Estudo

Trata-se de uma revisão de escopo conduzida com base no *Joana Briggs Institute Manual for Evidence Synthesis* ^(12)^ e nas recomendações de relato do *checklist Prisma Extension for Scoping Reviews* (PRISMA – ScR)^(13)^, a fim de garantir rigor metodológico, clareza e organização. O protocolo desta revisão foi cadastrado no OSF, sob DOI 10.17605/OSF.IO/57C2E. O estudo foi desenvolvido de acordo com as seguintes etapas: elaboração da pergunta de pesquisa, levantamento dos estudos relevantes, seleção, extração dos dados e apresentação dos resultados.

### Questão de Pesquisa

A formulação da pergunta de pesquisa foi baseada no acrônimo PCC (População/Conceito/Contexto), sendo a população (P) “indivíduos com feridas”, o conceito (C) “ferramentas de avaliação de infecção em feridas” e o contexto (C) “feridas infectadas”. Com essa combinação mnemônica, definiu-se a seguinte questão norteadora: Quais ferramentas disponíveis na literatura avaliam infecção em feridas?

### Critérios de Elegibilidade

Os critérios de inclusão para esta revisão de escopo abrangeram todos os manuscritos disponíveis na íntegra que respondessem à questão de pesquisa e estivessem alinhados ao objetivo do estudo. Para ampliar os resultados das buscas, não houve restrição quanto ao delineamento metodológico, período de publicação ou idioma. O contexto desta revisão foi amplo, sem restrição quanto ao cenário de assistência ou qualquer área específica de conhecimento. Foram considerados artigos publicados em periódicos e publicações da literatura cinzenta, como trabalhos de conclusão de curso, teses e dissertações.

Adotaram-se como ferramentas de avaliação de infecção para este estudo quaisquer constructos, instrumentos e tecnologias que visassem avaliar infecção em feridas. Quanto à etiologia, foram consideradas lesões com cicatrização por segunda intenção, tanto agudas (como queimaduras, feridas cirúrgicas e corto-contusas) quanto crônicas (como lesões por pressão, úlceras vasculares, neuropáticas e diabéticas). Foram excluídas da avaliação as feridas tumorais, por apresentarem uma patogênese específica e diferenciada das demais lesões.

### Estratégia de Busca

A estratégia de busca foi construída com base nos Descritores em Ciências da Saúde (DeCS) e *Medical Subject Headings* (MeSH), utilizando os operadores booleanos AND e OR para otimizar a recuperação de informações relevantes. As combinações foram elaboradas em conjunto com um bibliotecário, considerando as especificações de cada base de dados, a fim de abranger a máxima amplitude possível dos tópicos de interesse, garantindo, assim, uma coleta de dados robusta e abrangente, conforme visualizado no Quadro 1.

**Quadro 1:** Estratégia de buscas nas bases de dados – Rio de Janeiro, RJ, Brasil, 2024.

Estratégia de busca	Bases de dados/repositório	Artigos encontrados
(cicatrização) OR (cicatrização de feridas) OR (cicatrização de ferimentos) OR (wound healing) AND (estudo de avaliação) OR (estudos de avaliação) OR (evaluation study) OR (evaluation studies) OR (avaliação em enfermagem) OR (protocolos de enfermagem) OR (nursing assessment) OR (nursing protocols) AND (tool) OR (instruments) OR (ferramentas) OR (instrumentos) AND (infecção dos ferimentos) OR (infecção das feridas) OR (infecção da ferida) OR (infecção de feridas) OR (wound infection)	BVS	89
wound healing AND wound infection AND tool	CINAHL	112
wound healing AND evaluation study OR evaluation studies OR nursing assessment OR nursing protocols AND wound infection AND tool	PubMed	193
wound healing AND wound infection AND tool AND nursing	Scopus	40
“wound infection” AND “wound healing” AND “consensus document” OR “tools” AND “clinical practice”	Google Acadêmico	6.720

As bases de dados selecionadas incluíram o *Cumulative Index to Nursing and Allied Health Literature* (CINAHL), Scopus, a Biblioteca Virtual em Saúde (BVS) e o PubMed, devido à capacidade destas de reunir trabalhos de diversas áreas da saúde e da enfermagem, oferecendo uma ampla variedade de indexações. Essa escolha garantiu uma cobertura abrangente e diversificada da literatura disponível. Além disso, foi incluída a literatura cinzenta, conforme recomendado pela JBI, por meio do Google Acadêmico. As buscas ocorreram no período de 1º a 17 de maio de 2024. 

### Seleção dos Estudos

Após a pesquisa nas bases de dados, os resultados encontrados foram transferidos para o *software* Mendeley, onde foram realizadas a identificação e a remoção das duplicatas. Após a remoção dos trabalhos duplicados, procedeu-se à seleção dos artigos por meio da leitura dos títulos e resumos, com base nos critérios pré-estabelecidos no estudo. Os trabalhos pré-selecionados foram submetidos à leitura na íntegra para verificação da sua permanência e pertinência.

As informações dos documentos selecionados para análise foram extraídas por dois revisores, de maneira independente, com o uso de planilhas do Microsoft Excel^®^. Um terceiro revisor participou da validação das informações e da discussão para o estabelecimento de consenso entre os autores, quando necessário. O fluxo do processo de seleção dos manuscritos é mostrado em forma de diagrama, conforme as recomendações do PRISMA-ScR.

### Extração dos Dados

A extração de dados foi definida e adaptada de acordo com o manual do JBI, a fim de selecionar as seguintes informações relevantes: ano de publicação, fonte de extração da ferramenta ou título do manuscrito, visto que alguns trabalhos foram extraídos da mesma fonte, tipo de ferramenta de avaliação de infecção, etiologia da ferida e as principais características de infecção avaliadas pelas ferramentas.

### Apresentação dos Dados

Os dados extraídos foram apresentados em forma de quadro, com base nos dados categorizados. Foi realizada uma apresentação narrativa das informações, considerando o objetivo desta revisão de escopo. Para evidenciar as características clínicas mais comuns identificadas nos dados encontrados, elaborou-se uma nuvem de palavras utilizando-se o *site Word Art*. Esse recurso visual facilita a compreensão dos achados com maior frequência de aparecimento nos estudos.

### Aspectos Éticos

Esta revisão de escopo foi construída exclusivamente com base em literatura secundária disponível publicamente, sem envolver dados primários ou informações de identificação de indivíduos. Portanto, de acordo com as diretrizes éticas vigentes, não houve necessidade de submissão ao Comitê de Ética em Pesquisa, tampouco solicitação de Termo de Consentimento Livre e Esclarecido.

## RESULTADOS

De acordo com as estratégias de busca previamente estabelecidas, a pesquisa foi conduzida nas bases de dados e repositório acadêmico selecionados, resultando nos seguintes achados: 89 artigos na BVS, 112 na CINAHL, 193 na PubMed, 40 na Scopus e 6.720 no Google Acadêmico, totalizando 7.154 manuscritos. Em seguida, realizou-se a exclusão dos duplicados utilizando o *software* Mendeley, o que resultou na remoção de 44 documentos, deixando um total de 7.110 artigos para a leitura dos títulos e resumos.

Após a leitura e análise dos títulos e resumos, foram selecionados 116 manuscritos para leitura na íntegra, com base nos critérios de elegibilidade e na pergunta de pesquisa. Desses, 32 artigos foram finalmente incluídos na análise e discussão, conforme descrito no fluxograma do processo de busca e seleção apresentado na Figura 1.

**Figura 1:** Diagrama de fluxo PRISMA-ScR do processo de seleção das publicações da revisão. Rio de Janeiro, RJ, Brasil, 2024.

Após a análise dos artigos selecionados, foi confeccionado o Quadro 2, compilando as informações principais encontradas nos manuscritos selecionados, a fim de facilitar a apresentação clara e concisa dos dados. O quadro foi organizado e separado por ano de publicação, fonte ou título do manuscrito, tipo de ferramenta encontrada, nome da ferramenta, etiologia da ferida, características de infecção avaliadas pela ferramenta e país(es) dos estudos.

**Quadro 2:** Caracterização das publicações incluídas na revisão - Rio de Janeiro, RJ, Brasil, 2024.

	Ano/Fonte ou título do manuscrito	Tipo de ferramenta	Nome da ferramenta	Etiologia da ferida	Características de infecção avaliadas pela ferramenta	País(es) dos estudos
1	2023/Wound infection in clinical practice Principles of best practice do International Wound Infection Institute^(9)^	Sistema de Classificação	IDSA System	Úlcera diabética	Sinais e sintomas clínicos como: edema, eritema, exsudato purulento, dor, calor, além de alteração hemodinâmica.	Holanda, Austrália e Portugal
2	2023/Cuidados de enfermagem à pessoa com ferida em pé diabético: construção de instrumento^(14)^	Formulário de preenchimento	Instrumento para gerenciamento do cuidado de enfermagem à pessoa com pé diabético	Úlcera diabética	Sinais e sintomas clínicos como: exsudato purulento, dor, eritema, edema, odor fétido, calor, além de alteração hemodinâmica.	Brasil
3	2023/Validation of a Wound Tool for Assessment of Surgical Wounds in Infants^(15)^	Sistema de classificação	Ferramenta de avaliação de feridas cirúrgicas da Universidade de Alberta	Ferida cirúrgica	Sinais e sintomas clínicos como: exsudato purulento, presença de exsudato e alterações na pele ao redor da ferida (não específica quais alterações), necrose ou presença de prótese visível.	Canadá
4	2022/Development of a surgical wound assessment tool to measure healing and risk factors for delayed wound healing in Vietnam: a Delphi process^(16)^	Constructo de avaliação de feridas	SWAT	Ferida cirúrgica	Sinais e sintomas clínicos como: edema, eritema, exsudato, odor, dor, dentre outros sinais.	Vietnã
5	2022/Instrument for the evaluation of the chronic wounded patient: clinical, care and financial indicators^(17)^	Formulário de preenchimento	Instrumento para avaliação do paciente com ferida crônica da Universidade Federal de Minas Gerais	Feridas crônicas de diversas etiologias	Sinais e sintomas clínicos como: descolamento, necrose, exsudato, edema, endurecimento, odor, cor da pele ao redor da ferida (não especifica qual); sinais de colonização crítica no leito da lesão e sinais de infecção no leito da lesão (Estes últimos sem especificação).	Brasil
6	2022/Wound Infection in Clinical Practice -Principles of best practice^(9)^	Modelo conceitual	Wound Infection Continuum – International Wound Infection Institute (WIC – IWII)	Feridas agudas e crônicas de diversas etiologias	Sinais e sintomas clínicos como: hipergranulação; granulação friável; aumento do exsudato, cicatrização retarada, eritema, calor, edema, exsudato purulento, aumento da ferida, dor, odor, além de outros sinais e sintomas e alteração hemodinâmica.	EUA
7	2021/Post-dishcarge surveillance in surgical site infection: validation of an instrument^(18)^	Formulário de vigilância do sítio cirúrgicos pós-alta	Vigilância pós-alta para detecção de infecção de sítio cirúrgico da Universidade de São Paulo	Ferida cirúrgica	Sinais e sintomas clínicos como: exsudato purulento, edema, dor, calor, eritema, deiscência, dentre outros sinais e sintomas além de alteração hemodinâmica.	Brasil
8	2021/Contribution of software for recording, monitoring, and evaluating wounds^(19)^	Software	Wounds Monitoring	Feridas agudas e crônicas de diversas etiologias	Sinais e sintomas clínicos como: exsudato, sinais de inflamação e/ou infecção (não especifica quais), entre outros.	Brasil
9	2020/Validity of DMIST for monitoring healing of diabetic foot ulcers^(20)^	Escala	DMIST	Úlcera diabética	Sinais e sintomas clínicos como: endurecimento, exsudato purulento, odor desagradável, dentre outros sinais e sintomas além de alteração hemodinâmica.	Japão
10	2020/Therapeutic Index for Local Infections score validity: a retrospective European analysis^(21)^	Sistema de classificação	Therapeutic Index for Local Infections score	Úlcera de membros inferiores de diversas etiologias	Sinais e sintomas clínicos como: eritema, calor, edema, dor, cicatrização retardada, odor, exsudato purulento, dentre outros indicadores “não diretos de infecção”.	Alemanha
11	2020/Wound Infection in Clinical Practice -Principles of best practice^(9)^	Sistema de classificação	Infection Management Pathway	Feridas agudas e crônicas de diversas etiologias	Sinais e sintomas clínicos como: eritema, edema, exsudato purulento, dor, odor fétido, retardo na cicatrização, aumento do eritema, calor, hipergranulação, granulação friável, aumento da ferida, além de outros sinais e sintomas e alteração hemodinâmica.	Alemanha
12	2020/Validation of the Harikrishna Peripheral Skin Classification for wound assessment^(22)^	Sistema de classificação	Harikrishna Peripheral Skin Classification (HPSC)	Feridas agudas e crônicas de diversas etiologias	Classifica a pele peri lesional para infecção, através de sinais como: inflamação sem infecção, Inflamação com infecção, atípico – células senescentes/câncer/enfisema subcutâneo.	Malásia
13	2020/Management tools in nursing care for children with pressure injury^(23)^	Formulário de preenchimento e um Fluxograma	Instrumento de sistematização do cuidado de enfermagem à criança com lesão por pressão e Fluxograma de risco e prevenção de lesão por pressão da Universidade Federal do Espírito Santo	Lesão por pressão	Sinais e sintomas clínicos como: edema, aumento da temperatura, hiperemia e aumento de tecido necrosado.	Brasil
14	2019/Use of a bacterial fluorescence imaging device: wound measurement, bacterial detection and targeted debridement^(24)^	Dispositivo	MolecuLight	Úlceras diabéticas, venosas, arteriais, feridas operatórias e lesões por pressão	Avalia infecção através da carga bacteriana.	Canadá
15	2019/Evaluation of Wound Photography for Remote Postoperative Assessment of Surgical Site Infections^(25)^	Registro fotográfico através do uso de *smartphones*	Não se aplica	ISC (Infecção de Sítio Cirúrgico) ou OSC (Ocorrência de Sítio Cirúrgico)	Sinais de infecção relatados pelos pacientes como presentes ou ausentes e uso de imagens fotográficas, associadas ou não ao relato de infecção presente ou ausente.	EUA
16	2018/Importance of postprocedural Wound, Ischemia, and foot Infection (WIfI) restaging in predicting limb salvage^(26)^	Sistema de classificação	WIfI	Úlceras de membros inferiores de diversas etiologias	Sinais e sintomas clínicos como: infecção local envolvendo apenas a pele e o tecido subcutâneo, infecção local com eritema >2 cm ou envolvendo estruturas mais profundas que a pele e tecidos subcutâneos e infecção local com sinais de síndrome da resposta inflamatória sistêmica.	EUA
17	2018/Diagnostic value of fluorine-18 deoxyglucose positron emission tomography/computed tomography in deep sternal wound infection^(27)^	Tomografia computadorizada por emissão de pósitrons (PET/TC)	Não se aplica	Ferida cirúrgica	Sinais de infecção através da área comprometida demonstrada na imagem.	China
18	2018/Elaboration of an algorithm for wound evaluation and treatment^(28)^	Algoritmo	Algoritmo para avaliação e tratamento de feridas da Universidade do Vale do Sapucaí	Feridas agudas e crônicas de diversas etiologias	Sinais e sintomas clínicos como: calor, eritema, edema, dor, exsudato purulento, além de alteração hemodinâmica.	Brasil
19	2017/The inter-rater reliability between nurse-assessors clinically assessing infection of chronic wounds using the WUWHS criteria^(29)^	Formulário de preenchimento	WUWHS	Feridas crônicas de várias etiologias	Sinais e sintomas clínicos como: dor, cicatrização retardada, edema, tecido de granulação friável, odor fétido, descoloração, aumento do exsudato, endurecimento, eritema, além de alteração hemodinâmica.	Holanda
20	2017/Wound Infection in Clinical Practice -Principles of best practice^(9)^	Sistema de classificação	WIRE	Feridas agudas e crônicas de diversas etiologias	Sinais e sintomas clínicos como: dor, tecido necrótico, granulação friável, exposição de órgãos subjacentes, cicatrização retardada, eritema, calor, edema, odor, exsudato, dentre outros.	Reino Unido
21	2017/Inter-rater and intra-rater reliability outcomes of a rapid bacteria counting system with pressure ulcer samples^(30)^	Dispositivo Panasonic Healthcare DU-AA01NP-H	Não se aplica	Lesão por pressão	Avalia infecção através da carga bacteriana.	Japão
22	2016/The Wound Trend Scale: A Retrospective Review of Utility and Predictive Value in the Assessment and Documentation of Lower Leg Ulcers^(31)^	Escala	WTS	Úlceras de membros inferiores de diversas etiologias	Sinais e sintomas clínicos como: exsudato, tecido necrótico e desbridamento, profundidade, purulência, pele peri ferida, edema, dor e risco de infecção (eritema, calor, edema e dor, além de rastreio de infecção por exames).	Canadá
23	2016/Evaluation of a Surgical Site Discharge Teaching Tool Using Pictures and a Mirror^(32)^	Educação em saúde para ensino de autoavaliação de incisões cirúrgicas.	Não se aplica	Feridas cirúrgicas, especificamente após laparotomia	Sinais e sintomas clínicos como: calor, drenagem, exsudato purulento, eritema, edema, dor/sensibilidade, prurido, dentre outros sinais e sintomas.	EUA
24	2015/Adaptação cultural e validação para a população portuguesa de um instrumento de monitorização de feridas crónicas: escala RESVECH 2.0^(33)^	Escala	RESVECH 2.0	Feridas crônicas de várias etiologias	Sinais e sintomas clínicos como: dor; eritema, edema, calor, aumento de exsudato, exsudato purulento, tecido friável, ferida estagnada, tecido compatível com biofilme, odor, hiper granulação, aumento do tamanho da ferida, lesões satélites, palidez do tecido, dentre outros sinais e sintomas.	Portugal
25	2015/Construção de instrumento de coleta de dados para pessoas com feridas embasado na teoria de Wanda de Aguiar Horta^(34)^	Formulário de preenchimento	Instrumento de coleta de dados para pessoas com feridas embasado na teoria de Wanda de Aguiar Horta da Universidade Federal da Paraíba	Úlcera diabética	Sinais e sintomas clínicos como: edema, calor, eritema, aumento de tecido, dor e exsudato.	Brasil
26	2013/Reliability and validity of the Chinese version of DESIGN-R, an assessment instrument for pressure ulcers^(35)^	Formulário de preenchimento	DESIGN-R	Lesão por pressão	Sinais e sintomas clínicos, mas não específica quais, além de outras características gerais avaliadas da ferida como: profundidade da ferida, exsudato, tamanho, infecção, granulação e necrose.	China
27	2012/A systematic review of the ASEPSIS scoring system used in non-cardiac-related surgery^(36)^	Sistema de classificação	ASEPSIS	Feridas cirúrgicas	Sinais e sintomas clínicos, mas não específica quais, além de outras características gerais avaliadas da ferida como: secreção serosa, eritema, exsudato purulento, separação de tecidos profundos, isolamento de bactérias e internação por mais de 14 dias.	Reino Unido
28	2006/Wound Infection in Clinical Practice -Principles of best practice^(9)^	Sistema de classificação	NERDS and STONES	Feridas crônicas de várias etiologias	Sinais e sintomas clínicos como: não cicatrização das feridas, feridas exsudativas, tecido de granulação da superfície da ferida vermelho e sangrando, detritos na superfície da ferida, cheiro ou odor desagradável da ferida, aumento de tamanho, temperatura aumentada, sondagem ou exposição do osso, áreas de divisão novas ou lesões *satélites, e*xsudato, eritema, edema.	Canadá
29	2001/A tool to assess clinical signs and symptoms of localized infection in chronic wounds: development and reliability^(37)^	Lista de verificação	Clinical Signs and Symptoms Checklist (CSSC)	Feridas crônicas de diversas etiologias	Sinais e sintomas clínicos como: aumento de dor na área da úlcera, eritema, edema, calor, exsudato purulento, exsudato seroso associado a inflamação, retardo na cicatrização da úlcera, descoloração do tecido de granulação, tecido de granulação friável, odor fétido, feridas satélites e embolsamento na base da úlcera.	EUA
30	1997/The Sessing Scale for measurement of pressure ulcer healing^(38)^	Escala	Sessing Scale	Lesão por pressão	Sinais e sintomas clínicos como: drenagem purulenta, odor fétido, tecido necrótico, além de sintomas sépticos.	EUA
31	1997/Monitoring wound healing by odour^(39)^	Dispositivo com sensores	AromaScan	Úlcera vascular	Aroma das lesões e sua relação com os microrganismos presentes na ferida.	Reino Unido
32	1995/Wound registry: development and validation^(40)^	Formulário	Formulário de preenchimento da Universidade Estadual de Nova York	Feridas traumáticas	Sinais e sintomas clínicos como: Eritema, calor, sensibilidade ou drenagem, combinados com o julgamento clínico, além de avaliar aparência cosmética em curto tempo.	EUA

Para ajudar na identificação das principais características das infecções nos estudos analisados, foi criada uma nuvem de palavras, ilustrada na Figura 2. Essa representação visual destaca os principais sinais e sintomas clínicos de infecções em feridas. Os dados apresentados refletem as informações mais prevalentes encontradas nos trabalhos avaliados.

**Figura 2:** Nuvem de palavras dos sinais e sintomas frequentes nas publicações. Rio de Janeiro, RJ, Brasil, 2024.

Os achados desta revisão identificaram 32 ferramentas para avaliação de infecção em feridas, com publicações variando de 1995 a 2023, sendo 13 trabalhos publicados nos últimos 5 anos. A maioria das ferramentas encontradas avalia a infecção de forma qualitativa, por meio dos sinais e sintomas clínicos, correspondendo a 26 instrumentos^(9, 14, 15, 16, 17, 18, 20, 21, 22, 23, 26, 28, 29, 31, 32, 33, 34, 35, 36, 37, 38, 40, 41, 42)^. As demais avaliam por meio de recursos tecnológicos mais sofisticados, como equipamentos e dispositivos eletrônicos, totalizando 5 ferramentas, e apenas 1 utiliza *software* para avaliação da lesão e infecção^(19, 24, 25, 27, 30, 39)^.

As ferramentas que avaliam a infecção por meio dos sinais e sintomas apresentados pela ferida são constructos tradicionais, que se apresentam de diversas formas, variando entre formulários, escalas, sistemas de classificação, *checklists*, acrônimos, cartilhas explicativas e algoritmo, podendo ser impressas em papel para preenchimento a caneta ou em plataformas digitais. Tais ferramentas avaliam a infecção por meio da observação direta da pele, leito da lesão e da pele perilesional. Os sinais e sintomas de infecção avaliados variam conforme as especificidades de cada constructo e da etiologia da ferida avaliada^(9, 14, 15, 16, 17, 18, 20, 21, 22, 23, 26, 28, 29, 31, 32, 33, 34, 35, 36, 37, 38, 40, 41, 42)^.

Os instrumentos que avaliam infecções em feridas por meio de recursos tecnológicos incluem equipamentos que utilizam imagens por emissão de pósitrons combinadas com tomografia computadorizada, avaliação quantitativa da carga microbiana por meio de imagens de imunofluorescência, *softwares* para registro, monitoramento e análise de feridas (incluindo sinais e sintomas de infecção), sensores para avaliação do odor como indicador preditivo de infecção e, ainda, o uso de *smartphones* para auxiliar na identificação de complicações em feridas, como infecções^(19, 24, 25, 27, 30, 39)^.

Entre as ferramentas tradicionais que avaliam infecção com base em sinais e sintomas clínicos, destacam-se o *Clinical Signs and Symptoms Checklist* (CSSC), *Therapeutic Index for Local Infections* (TILI), *Infection Management Pathway, Wound Infection Continuum - International Wound Infection Institute* (WIC- IWII), *World Union of Wound Healing Societies* (WUWHS) e os acrônimos ASEPSIS, WIRE, NERDS e STONES, WIfI, IWGDF/IDSA *System* ^(9, 21, 26, 29, 36, 37, 41, 42)^. Outros instrumentos também foram identificados nos estudos analisados, embora não possuam nomes específicos^(14, 15, 16, 17, 18, 23, 28, 32, 34, 40)^. Os sinais e sintomas clássicos de infecção mencionados na maioria dessas ferramentas incluem dor, calor, edema, eritema, exsudato purulento e odor^(9, 14, 15, 16, 17, 18, 20, 21, 22, 23, 26, 28, 29, 31, 32, 33, 34, 35, 36, 37, 38, 40, 41, 42)^.

Apenas os acrônimos NERDS e STONES definiram a quantidade de sinais e sintomas necessários para classificar a infecção em uma ferida^(9)^. Nenhuma outra ferramenta especificou a quantidade de características clínicas que devem estar presentes na lesão para o diagnóstico de infecção^(9, 21, 26, 29, 36, 37, 41, 42)^. Conforme apontado pelas ferramentas tradicionais, nenhum sinal ou sintoma único determina de forma confiável a presença ou ausência de infecção em uma ferida. Assim, a combinação de múltiplos sinais e sintomas indicativos de infecção deve ser utilizada para um diagnóstico clínico mais preciso e seguro^(9, 29)^.

Os instrumentos também identificaram que alguns sinais e sintomas são mais específicos e sensíveis como preditores de infeção do que outros. Além disso, certos sinais podem manifestar-se de forma sutil ou até mesmo não aparecer, dependendo do tipo de ferida e das condições clínicas do paciente^(9, 34, 37, 41)^. Os trabalhos demonstraram ainda que os sinais de inflamação – calor, eritema, edema e dor – podem estar presentes em feridas infectadas, sendo considerados como sinais clássicos de infecção^(9, 14, 15, 16, 19, 20, 23, 24, 27, 30, 31, 32, 33, 34, 36, 39, 40)^.

Outras ferramentas analisadas possuem, em sua composição, um item ou domínio que avalia infecção, entre outras características, embora não tenham sido projetadas especificamente para esse propósito. Entre essas ferramentas estão o RESVECH 2.0, DESIGN-R, *Harikrishna Periwound Skin Classification* (HPSC), *Wound Trend Scale* (WTS), *Sessing Scale*, os mneumônicos DMIST e SWAT, além de outras ferramentas sem nome específico. Os sinais e sintomas clínicos de infecção mais frequentemente identificados incluem: exsudato purulento, odor fétido, eritema, edema^(16, 20, 22, 31, 33, 35, 38)^.

No que se refere às ferramentas que avaliam quantitativamente a carga microbiana presente no leito da lesão e sua concentração, destacam-se o dispositivo Molecu*Light* e o aparelho Panasonic *Healthcare* DU-AA01NP-H. Esses dispositivos permitem a avaliação da infecção a beira leito de forma menos invasiva e rápida^(24, 30)^. Além disso, o equipamento AromaScan, auxilia no monitoramento do progresso da ferida e na identificação de possível infecção com base no padrão de odores detectados. Sensores de polímeros condutores, por sua vez, apresentam mudanças na resistência elétrica quando expostos a mistura de produtos químicos voláteis^(39)^.

O uso de *smartphones*, também encontrado no mapeamento das ferramentas desta pesquisa, pode ser empregado para auxiliar na avaliação de complicações em feridas operatórias, como ISC (Infecção de Sítio Cirúrgico) ou OSC (Ocorrência de Sítio Cirúrgico), incluindo seroma, celulite, hematoma, deiscência e abscesso^(25)^. Quanto à tomografia computadorizada, essa tecnologia mostrou-se como uma possibilidade de identificação de infecção em estruturas mais profundas da ferida^(27)^.

Em relação à distribuição geográfica das ferramentas, foram identificadas 18 no continente americano, sendo 7 no Brasil, 4 no Canadá e 7 nos Estados Unidos^(9, 14, 15, 17, 18, 19, 23, 24, 24, 25, 26, 28, 31, 32, 34, 37, 38, 40)^. Na Ásia, foram encontradas 6 ferramentas: 1 no Vietnã, 2 no Japão, 2 na China e 1 na Malásia^(16, 20, 22, 30, 35)^. Na Europa, foram localizadas 8 ferramentas, distribuídas entre Alemanha (2), Holanda (2), Reino Unido (3) e Portugal (1)^(9, 21, 29, 33, 36)^. Nos demais continentes, não foram identificados trabalhos publicados nesta revisão de escopo.

Dentre as dez ferramentas clínicas tradicionais, específicas para avaliação de infecção por meio de sinais e sintomas, 6 são do continente europeu^(9, 21, 29, 36)^ e 4 do continente americano^(9, 26, 37)^. As demais apresentam algum item ou domínio de avaliação de infecção, mas não são específicas para este fim^(14, 15, 16, 17, 18, 19, 20, 22, 23, 28, 31, 32, 33, 34, 35, 38, 40)^. Nenhum dos estudos brasileiros mapeados nesta revisão avalia exclusivamente o processo infeccioso em feridas^(14, 17, 18, 19, 23, 28, 34)^. 

## DISCUSSÃO

O mapeamento das evidências disponíveis na literatura revela que as ferramentas de avaliação de infecção em feridas apresentam diferentes aplicabilidades, com predomínio de instrumentos baseados em abordagens tradicionais, que avaliam a infecção por meio de sinais e sintomas clínicos observados nas lesões. Esse achado está alinhado às principais diretrizes internacionais, que recomendam a análise das características da ferida para o diagnóstico de infecção. Tal abordagem destaca-se por ser prática, acessível, rápida e viável para aplicação em diferentes cenários clínicos, além de apresentar boa validade e confiabilidade, possibilitando um diagnóstico precoce^(9, 14, 15, 16, 17, 18, 20, 21, 22, 23, 26, 28, 29, 31, 32, 33, 34, 35, 36, 37, 38, 40, 41, 42,)^.

Os sinais clínicos desempenham um papel fundamental no monitoramento da evolução de feridas infectadas, sendo elementos-chave para orientar decisões terapêuticas e avaliar a resposta ao tratamento^(8, 10, 29, 31, 33)^. As ferramentas de avaliação permitem a organização desses sinais de forma sistematizada, promovendo um acompanhamento estruturado e mais eficiente^(7, 9, 11)^. A análise criteriosa dos sinais clínicos auxilia os profissionais de saúde a identificar alterações precoces no estado da lesão, possibilitando ajustes oportunos nas intervenções terapêuticas e contribuindo para a melhoria dos desfechos clínicos^(8, 10)^.

A maioria das ferramentas que avaliam infecção com base em sinais e sintomas clínicos inclui os sinais cardinais da inflamação: dor, calor, edema e eritema. Isso se justifica pelo fato de que o processo inflamatório está presente em feridas infectadas, tornando esses sinais também indicadores relevantes de infecção. Sabe-se que não são específicos de infecção se avaliados isoladamente, mas devem ser considerados como preditivos de infecção em feridas que apresentam essas características sem uma causa identificada. De acordo com algumas ferramentas, os sinais e sintomas de infecção ainda se classificam em sinais clássicos, sutis e sistêmicos^(8, 9, 14, 20, 21, 26, 29, 41, 42)^.

Ferramentas tecnológicas para a avaliação de infecção em feridas são menos frequentes na literatura, possivelmente devido ao custo elevado e à disponibilidade limitada^(24, 27, 30, 39)^. No entanto, essas tecnologias podem ser particularmente úteis em contextos que exigem maior precisão e agilidade. Os estudos revisados sugerem a necessidade de mais pesquisas para consolidar o uso dessas tecnologias na prática clínica. Apesar disso, elas já demonstram potencial para oferecer um diagnóstico rápido, seguro e menos invasivo, favorecendo o tratamento em tempo oportuno e o manejo efetivo da infecção^(9, 19, 24, 25, 27, 30, 39)^.

Quanto aos tipos de feridas avaliadas, observa-se que há diversidade de etiologias, abrangendo tanto feridas agudas quanto crônicas, o que possibilita sua aplicação em diversos contextos de cuidado, como atenção básica, domiciliar e hospitalar^(9, 21, 26, 29, 36, 37, 41, 42)^. Esse achado é semelhante aos resultados de Cardinelli et al.^(11)^, que também identificaram instrumentos de avaliação aplicáveis a diferentes etiologias e cenários clínicos.

A literatura apresenta uma ampla gama de instrumentos para a avaliação de feridas infectadas, que variam desde ferramentas abrangentes, capazes de avaliar múltiplas lesões, até outras mais específicas para determinadas etiologias^(9, 14, 15, 16, 17, 18, 20, 21, 22, 23, 26, 28, 29, 31, 32, 33, 34, 35, 36, 37, 38, 40, 41, 42)^. Conhecer esses instrumentos e suas particularidades é essencial para que os profissionais de saúde possam adaptar os cuidados de forma mais precisa, segura e eficaz^(11)^.

A utilização de ferramentas para avaliação de feridas tem se mostrado uma tecnologia capaz de melhorar a qualidade do atendimento aos pacientes, uma vez que orienta a prática clínica no tratamento e gerenciamento dessas lesões com base nas melhores evidências, além de seu uso permitir uma padronização da análise das feridas, reduzindo a subjetividade na avaliação. Embora a literatura aponte esses instrumentos como uma possibilidade de melhoria da qualidade dos cuidados, há poucos estudos publicados que comprovem a efetividade de seu uso rotineiro e sistematizado na prática clínica^(11, 43)^.

A aplicação dessas tecnologias no manejo de pacientes com feridas pode apresentar benefícios significativos, logo, é necessária uma minuciosa avaliação para escolha adequada, a depender do contexto em que as lesões são gerenciadas e o objetivo da avaliação. Além disso, é fundamental compreender como utilizar essas ferramentas corretamente, para evitar seu uso inadequado e, consequentemente, avaliações equivocadas^(11, 43)^.

No que diz respeito à avaliação de infecção, o consenso do *International Wound Infection Institute* (IWII), formulado em 2022, apresenta ferramentas específicas para a identificação de infecções em feridas de diversas etiologias. Algumas dessas estão em fase de validação psicométrica, enquanto outras já foram validadas para uso clínico. Além disso, elas incluem a análise de fatores de risco para infecção^(9)^.

No contexto brasileiro, uma revisão de escopo mapeou 51 instrumentos disponíveis na literatura para avaliação de feridas, dos quais apenas oito foram adaptados e validados para uso no Brasil. No entanto, nenhum desses instrumentos é específico para infecções em feridas. A maioria das ferramentas traduzidas para o português foca na avaliação do processo de cicatrização, na classificação das lesões e no preparo adequado do leito da ferida para otimizar o tratamento^(11)^.

Além da variedade de instrumentos disponíveis, é crucial considerar a validade e a confiabilidade das ferramentas de avaliação. Não existe uma ferramenta considerada “padrão-ouro” para a identificação de infecções em feridas, pois essa escolha depende do tipo de lesão, do cenário de avaliação e dos recursos disponíveis. Ademais, a equipe de saúde deve ser devidamente treinada e habilitada para utilizar as ferramentas de avaliação de infecções de forma segura e eficaz. Independentemente do instrumento escolhido, sabe-se que uma ferramenta confiável e validada auxilia no cuidado de pacientes com infecções em feridas, prevenindo complicações e promovendo uma melhor tomada de decisão^(9, 11, 43)^.

Apesar dos avanços na ciência e tecnologia aplicada ao cuidado com feridas, ainda há uma lacuna significativa na sistematização de avaliação e validação de ferramentas específicas para identificar infecções em feridas. Essa revisão de escopo busca contribuir para preencher essa lacuna ao mapear as ferramentas existentes, proporcionando um panorama abrangente de suas características. Além disso, a pesquisa evidencia a falta de instrumentos validados no contexto brasileiro para avaliação de infecção.

Os resultados desta revisão fornecem uma base para futuras investigações clínicas e estudos metodológicos voltados à criação e validação de ferramentas, fortalecendo a prática baseada em evidências. Ao sistematizar os dados, esta revisão busca auxiliar os profissionais de saúde na escolha de métodos apropriados para diagnosticar e manejar infecções em feridas, promovendo a padronização dos cuidados e, consequentemente, melhores desfechos clínicos para os pacientes.

## CONCLUSÃO

A diversidade de ferramentas para avaliação de infecção em feridas disponível na literatura reflete a complexidade e multidimensionalidade do gerenciamento de feridas infectadas. Esses instrumentos variam desde formulários simples para preenchimento manual e avaliação global da lesão, abrangendo diferentes aspectos como a presença de sinais e sintomas clínicos de infecção, até o uso de equipamentos mais avançados e tecnológicos para análise de carga microbiana e exames de imagens.

As ferramentas de avaliação mais utilizadas na prática clínica baseiam-se em sinais clínicos, e não existem instrumentos validados no Brasil para identificar especificamente infecções em feridas. Este estudo sugere o desenvolvimento de ferramentas adaptadas à realidade brasileira e a realização de mais pesquisas para validar a confiabilidade, os benefícios e as limitações desses instrumentos na prática assistencial.

# Translation: Herramientas de evaluación de infecciones para heridas agudas y crónicas: una revisión del alcance

**DOI:** 10.1590/1980-220X-REEUSP-2024-0392es

**Authors:** Daniella Cristina Julio Lima, Graciele Oroski Paes

**Affiliations:** 1 Universidade Federal do Rio de Janeiro, Escola de Enfermagem Anna Nery, Departamento de Enfermagem Fundamental, Rio de Janeiro, RJ, Brasil.

**Keywords:** Cicatrización de Heridas, Medición de Riesgo, Infección de Heridas, Técnicas y Procedimientos Diagnósticos, Enfermería

## Abstract

**Objetivo:**

Mapear las herramientas disponibles en la literatura para evaluar la infección de heridas.

**Método:**

Se trata de una revisión de alcance realizada desde el *Joana Briggs Institute Manual for Evidence Synthesis*, en las bases de datos de la Biblioteca Virtual en Salud, PubMed, Scopus, *Cumulative Index to Nursing and Allied Health Literature* y el repositorio de literatura académica, Google Scholar. La pregunta de investigación se desarrolló con base en el acrónimo PCC (Población/Concepto/Contexto), dando como resultado la siguiente formulación: “¿Qué herramientas disponibles en la literatura evalúan la infección de la herida?”

**Resultados:**

El análisis incluyó 32 herramientas de evaluación de infecciones de heridas. De estos, se identificaron 26 que utilizan signos y síntomas clínicos de infección distribuidos en escalas, listas de verificación, sistemas de clasificación y formularios de llenado. Además de estos, se identificaron 5 dispositivos electrónicos y 01 software.

**Conclusión:**

La diversidad de instrumentos resalta la complejidad del manejo de las infecciones de heridas, enfatizando la necesidad de discutir su aplicabilidad, beneficios y limitaciones para seleccionar el mejor instrumento basado en evidencia.

## INTRODUCCIÓN

El tratamiento de las heridas infectadas representa un reto para los profesionales sanitarios debido a la complejidad que entraña su gestión. Este proceso incluye la comprensión de los factores biopsicosociales y ambientales que interfieren en el cuidado de las personas con heridas agudas y crónicas. Además, se requiere un amplio conocimiento de los diversos factores que influyen en el proceso de cicatrización, de las opciones terapéuticas disponibles, que varían en función de la accesibilidad y disponibilidad de recursos, y de la necesidad de realizar un seguimiento periódico de estos pacientes^(1, 2, 3)^.

La presencia de una herida, especialmente las heridas crónicas como las úlceras por presión, las úlceras neuropáticas, las úlceras diabéticas y las úlceras vasculares, tiene un impacto negativo en la calidad de vida de los pacientes. Estas lesiones suelen causar dolor, limitaciones y aislamiento social, interfiriendo en las actividades de la vida diaria^(3)^. Pueden derivar en complicaciones graves, como osteomielitis y sepsis, e incluso provocar la muerte. Además, ocasionan elevados costes para el sistema sanitario debido a la prolongación del tratamiento y la hospitalización en algunos casos^(1, 2)^.

A pesar de las recomendaciones y guías existentes para el tratamiento y manejo de heridas de diversas etiologías, no existe consenso en la literatura sobre la mejor práctica a aplicar de forma sistematizada y estandarizada en la asistencia. Muchos profesionales siguen enfrentándose a la variabilidad en la aplicación de las mejores pruebas y tienen dificultades para identificar y evaluar de forma coherente la gravedad de las infecciones^(4, 5)^. En la práctica clínica, el tratamiento de las infecciones de heridas se basa en gran medida en las experiencias y conocimientos de los profesionales sanitarios, a menudo sin especialización en el área del cuidado de heridas^(4, 6)^.

La identificación de la infección se realiza tradicionalmente mediante la observación clínica y visual de las heridas, sin que exista una sistematización o estandarización en la evaluación. El diagnóstico de la infección es complejo porque las características de la herida varían según la etiología y la morbilidad del paciente, lo que dificulta la identificación del proceso infeccioso en algunos casos, especialmente en las heridas crónicas, que pueden ser sutiles o incluso asintomáticas^(6, 7)^. La falta de herramientas estructuradas que ayuden a esta identificación y a la toma de decisiones da lugar a un enfoque a menudo subjetivo y dependiente de la experiencia del profesional sanitario^(8, 9)^.

En la literatura, existen pocos estudios epidemiológicos sobre la prevalencia e incidencia de heridas infectadas, especialmente en Brasil. La mayoría de los estudios existentes en el contexto brasileño apuntan a resultados puntuales, generalmente restringidos a niveles institucionales, sin un sistema de monitoreo específico integrado a las redes de salud, además de mostrar alta variabilidad dependiendo de la región del país, del tipo de estudio y de la etiología de la herida analizada^(7)^.

El uso de herramientas puede ser una estrategia adoptada en las unidades que tratan heridas como forma de estandarizar su manejo y tratamiento, permitiendo una valoración más objetiva y sistematizada. Esto ayuda y orienta al profesional en una evaluación estructurada, utilizando instrumentos validados y fiables, especialmente cuando se trata de identificar y evaluar infecciones de heridas^(7, 8)^. Se sabe que el uso inadecuado e indiscriminado de antimicrobianos puede dar lugar a resistencias microbianas. Por ello, el uso de herramientas para estas afecciones permite identificar precozmente las infecciones y tratarlas a tiempo^(9)^.

Por lo tanto, una evaluación precisa y eficaz para la identificación de la infección de la herida es esencial para garantizar un tratamiento adecuado y la prevención de complicaciones posteriores. Como se ha explicado anteriormente, el uso de herramientas para la evaluación de la infección de heridas sirve de ayuda a los profesionales sanitarios en la identificación adecuada de la infección, en la toma de decisiones para un tratamiento oportuno y seguro, en la gestión eficaz y eficiente de la lesión, así como en una evaluación sistematizada y organizada^(7, 10, 11)^.

Se realizó una búsqueda bibliográfica preliminar entre abril y mayo de 2024, en la que no se identificó ninguna revisión sistemática o de alcance dirigida a mapear herramientas para evaluar la infección de heridas. Tampoco se encontraron registros de estudios en curso en las plataformas PROSPERO, *Open Science Framework* (OSF) o *Figshare*. La falta de estudios que ofrezcan una visión global y sistematizada de las herramientas disponibles pone de manifiesto una importante laguna en la bibliografía y justifica esta investigación.

Esta laguna en la bibliografía, unida a las importantes repercusiones de las infecciones de heridas sobre las personas y los sistemas sanitarios -como estancias hospitalarias más prolongadas, aumento de los costes de tratamiento, riesgo de complicaciones graves y mortalidad^(1)^ - pone de manifiesto la relevancia clínica y asistencial del tema. Este estudio, esencial y sin precedentes, tiene como objetivo trazar un mapa de las herramientas disponibles en la bibliografía para evaluar la infección de heridas.

## MÉTODO

### Tipo de Estudio

Se trata de una revisión de alcance basada en el *Manual para la Síntesis de la Evidencia del Instituto Joana Briggs* ^(12)^y en las recomendaciones para la elaboración de informes de la *lista de verificación Prisma Extension for Scoping Reviews* (PRISMA – ScR)^(13)^ con el fin de garantizar el rigor metodológico, la claridad y la organización. El protocolo de esta revisión se registró en el OSF con el DOI ١٠.١٧٦٠٥/OSF.IO/٥٧C٢E. El estudio se realizó de acuerdo con las siguientes etapas: elaboración de la pregunta de investigación, encuesta de estudios relevantes, selección, extracción de datos y presentación de los resultados.

### Pregunta de Investigación

La formulación de la pregunta de investigación se basó en el acrónimo PCC (Población/Concepto/Contexto), siendo la población (P) «individuos con heridas», el concepto (C) «herramientas de evaluación de la infección de heridas» y el contexto (C) «heridas infectadas». Con esta combinación mnemotécnica, se definió la siguiente pregunta guía: ¿Qué herramientas disponibles en la literatura evalúan la infección de heridas?

### Criterios de Inclusión

Los criterios de inclusión para esta revisión exhaustiva abarcaron todos los manuscritos completos disponibles que respondieran a la pregunta de investigación y estuvieran en consonancia con el objetivo del estudio. Con el fin de ampliar los resultados de la búsqueda, no hubo restricciones en cuanto al diseño metodológico, el período de publicación o el idioma. El contexto de esta revisión fue amplio, sin restricciones en cuanto al ámbito asistencial ni a ningún área de conocimiento específica. Se consideraron artículos publicados en revistas y literatura gris, como trabajos de finalización de curso, tesis y tesinas.

Para este estudio se adoptaron como herramientas de evaluación de la infección todos los constructos, instrumentos y tecnologías destinados a evaluar la infección de heridas. En cuanto a la etiología, se consideraron las heridas con cicatrización por segunda intención, tanto agudas (como quemaduras, heridas quirúrgicas y contusiones por corte) como crónicas (como lesiones por presión, úlceras vasculares, neuropáticas y diabéticas). Se excluyeron de la evaluación las heridas tumorales, ya que tienen una patogenia específica que difiere de otras lesiones.

### Estrategia de Búsqueda

La estrategia de búsqueda se basó en los Descriptores de Ciencias de la Salud (DeCS) y *los Encabezamientos de Materias Médicas* (MeSH), utilizando los operadores booleanos AND y OR para optimizar la recuperación de información relevante. Las combinaciones se elaboraron conjuntamente con un bibliotecario, teniendo en cuenta las especificaciones de cada base de datos con el fin de abarcar la mayor variedad posible de temas de interés, garantizando así una recopilación de datos sólida y exhaustiva, como se muestra en el Cuadro 1.

**Cuadro 1:** Estrategia de búsqueda en bases de datos – Río de Janeiro, RJ, Brasil, 2024.

Estrategia de búsqueda	Bases de datos/epositorio	Artículos encontrados
(cicatrização) OR (cicatrização de feridas) OR (cicatrização de ferimentos) OR (wound healing) AND (estudo de avaliação) OR (estudos de avaliação) OR (evaluation study) OR (evaluation studies) OR (avaliação em enfermagem) OR (protocolos de enfermagem) OR (nursing assessment) OR (nursing protocols) AND (tool) OR (instruments) OR (ferramentas) OR (instrumentos) AND (infecção dos ferimentos) OR (infecção das feridas) OR (infecção da ferida) OR (infecção de feridas) OR (wound infection)	BVS	89
wound healing AND wound infection AND tool	CINAHL	112
wound healing AND evaluation study OR evaluation studies OR nursing assessment OR nursing protocols AND wound infection AND tool	PubMed	193
wound healing AND wound infection AND tool AND nursing	Scopus	40
"wound infection" AND "wound healing" AND "consensus document" OR "tools" AND "clinical practice"	Google Acadêmico	6.720

Las bases de datos seleccionadas incluyeron el *Cumulative Index to Nursing and Allied Health Literature*(CINAHL), Scopus, la Biblioteca Virtual de Salud (BVS) y PubMed, debido a su capacidad para reunir trabajos de diversas áreas de la salud y la enfermería, ofreciendo una amplia variedad de indización. Esta elección garantizó una cobertura amplia y diversa de la literatura disponible. Además, se incluyó literatura gris, tal y como recomienda el JBI, a través de Google Scholar. Las búsquedas se realizaron entre el 1 y el 17 de mayo de 2024.

### Selección de Estudios

Tras la búsqueda en las bases de datos, los resultados encontrados se transfirieron al programa Mendeley, donde se identificaron y eliminaron los duplicados. Una vez eliminados los duplicados, se seleccionaron los artículos mediante la lectura de los títulos y resúmenes, basándose en los criterios de estudio preestablecidos. Los artículos preseleccionados se leyeron en su totalidad para comprobar su permanencia y pertinencia.

La información de los documentos seleccionados para el análisis fue extraída de forma independiente por dos revisores mediante hojas de cálculo de Microsoft Excel^®^. Un tercer revisor participó en la validación de la información y en la discusión para establecer un consenso entre los autores, cuando fue necesario. El flujo del proceso de selección de manuscritos se muestra en forma de diagrama, de acuerdo con las recomendaciones PRISMA-ScR.

### Extracción de Datos

La extracción de datos se definió y adaptó según el manual del JBI para seleccionar la siguiente información relevante: año de publicación, fuente de extracción de la herramienta o título del manuscrito, ya que algunos trabajos se extrajeron de la misma fuente, tipo de herramienta de evaluación de la infección, etiología de la herida y principales características de la infección evaluada por las herramientas.

### Presentación de Los Datos

Los datos extraídos se presentaron en forma de tabla, basándose en los datos categorizados. Se realizó una presentación narrativa de la información, teniendo en cuenta el objetivo de esta revisión de alcance. Para destacar las características clínicas más comunes identificadas en los datos encontrados, se creó una nube de palabras utilizando el *sitio web Word Art*. Este recurso visual facilita la comprensión de los hallazgos que aparecen con mayor frecuencia en los estudios.

### Aspectos Éticos

Esta revisión exploratoria se construyó exclusivamente a partir de literatura secundaria disponible públicamente, sin involucrar datos primarios o información que identifique a individuos. Por lo tanto, de acuerdo con las directrices éticas vigentes, no hubo necesidad de someterla al Comité de Ética de la Investigación, ni se solicitó un Formulario de Consentimiento Informado.

## RESULTADOS

De acuerdo con las estrategias de búsqueda previamente establecidas, la investigación se realizó en las bases de datos y repositorios académicos seleccionados, obteniéndose los siguientes resultados: 89 artículos en BVS, 112 en CINAHL, 193 en PubMed, 40 en Scopus y 6.720 en Google Scholar, con un total de 7.154 manuscritos. A continuación, se excluyeron los duplicados utilizando el *programa* Mendeley, lo que dio lugar a la eliminación de ٤٤ documentos, dejando un total de ٧.١١٠ artículos para la lectura de los títulos y resúmenes.

Tras la lectura y el análisis de los títulos y resúmenes, se seleccionaron 116 manuscritos para su lectura completa, en función de los criterios de elegibilidad y la pregunta de investigación. De ellos, 32 artículos se incluyeron finalmente en el análisis y la discusión, como se describe en el diagrama de flujo del proceso de búsqueda y selección que se muestra en la Figura 1.

**Figura 1:** Diagrama de flujo PRISMA-ScR del proceso de selección de publicaciones de revisión. Rio de Janeiro, RJ, Brasil, 2024.

Tras el análisis de los artículos seleccionados, se elaboró la Cuadro 2, que recopila las principales informaciones encontradas en los manuscritos seleccionados, con el fin de facilitar una presentación clara y concisa de los datos. Lo cuadro se organizó y separó por año de publicación, fuente o título del manuscrito, tipo de herramienta encontrada, nombre de la herramienta, etiología de la herida, características de la infección evaluadas por la herramienta y país(es) de los estudios.

**Cuadro 2:** Caracterización de las publicaciones incluidas en la revisión – Rio de Janeiro, RJ, Brasil, 2024.

	Año/Fuente o título del manuscrito	Tipo de herramienta	Nombre de la herramienta	Etiología de la herida	Características de infección evaluadas por la herramienta	País(es) de los estudios
1	2023/Wound infection in clinical practice Principles of best practice do International Wound Infection Institute^(9)^	Sistema de Clasificación	IDSA System	Úlcera diabética	Signos y síntomas clínicos como edema, eritema, exudado purulento, dolor, calor y cambios hemodinámicos.	Holanda, Australia y Portugal
2	2023/Cuidados de enfermagem à pessoa com ferida em pé diabético: construção de instrumento^(14)^	Formulario de inscripción	Una herramienta para gestionar los cuidados de enfermería a personas con pie diabético	Úlcera diabética	Signos y síntomas clínicos como exudado purulento, dolor, eritema, edema, mal olor, calor, así como cambios hemodinámicos.	Brasil
3	2023/Validation of a Wound Tool for Assessment of Surgical Wounds in Infants^(15)^	Sistema de clasificación	Herramienta de evaluación de heridas quirúrgicas de la Universidad de Alberta	Herida quirúrgica	Signos y síntomas clínicos como exudado purulento, presencia de exudado y cambios en la piel alrededor de la herida (no se especifica qué cambios), necrosis o presencia de prótesis visibles	Canadá
4	2022/Development of a surgical wound assessment tool to measure healing and risk factors for delayed wound healing in Vietnam: a Delphi process^(16)^	Constructo de evaluación de heridas	SWAT	Herida quirúrgica	Signos y síntomas clínicos como edema, eritema, exudado, olor, dolor, entre otros signos.	Vietnam
5	2022/Instrument for the evaluation of the chronic wounded patient: clinical, care and financial indicators^(17)^	Formulario de inscripción	Instrumento de evaluación de pacientes con heridas crónicas de la Universidad Federal de Minas Gerais	Heridas crónicas de diversas etiologías	Signos y síntomas clínicos como: desprendimiento, necrosis, exudado, edema, induración, olor, color de la piel alrededor de la herida (no especifica cuáles); signos de colonización crítica en el lecho de la lesión y signos de infección en el lecho de la lesión (estos últimos sin especificar).	Brasil
6	2022/Wound Infection in Clinical Practice -Principles of best practice^(9)^	Modelo conceptual	Wound Infection Continuum – International Wound Infection Institute (WIC – IWII)	Heridas agudas y crónicas de diversas etiologías	Signos y síntomas clínicos como: hipergranulación; granulación friable; aumento del exudado, retraso de la cicatrización, eritema, calor, edema, exudado purulento, agrandamiento de la herida, dolor, olor, así como otros signos y síntomas y cambios hemodinámicos.	EUA
7	2021/Post-dishcarge surveillance in surgical site infection: validation of an instrument^(18)^	Formulário de vigilância do local cirúrgico após a alta	Vigilancia post-alta de infecciones del sitio quirúrgico en la Universidad de São Paulo	Herida quirúrgica	Signos y síntomas clínicos como: exudado purulento, edema, dolor, calor, eritema, dehiscencia, entre otros signos y síntomas, así como cambios hemodinámicos.	Brasil
8	2021/Contribution of software for recording, monitoring, and evaluating wounds^(19)^	Software	Wounds Monitoring	Heridas agudas y crónicas de diversas etiologías	Signos y síntomas clínicos como exudado, signos de inflamación y/o infección (no especifica cuál), entre otros.	Brasil
9	2020/Validity of DMIST for monitoring healing of diabetic foot ulcers^(20)^	Escala	DMIST	Úlcera diabética	Signos y síntomas clínicos como endurecimiento, exudado purulento, olor desagradable, entre otros signos y síntomas, así como cambios hemodinámicos.	Japón
10	2020/Therapeutic Index for Local Infections score validity: a retrospective European analysis^(21)^	Sistema de clasificación	Therapeutic Index for Local Infections score	Úlceras de miembros inferiores de diversas etiologías	Signos y síntomas clínicos como: eritema, calor, edema, dolor, retraso en la cicatrización, olor, exudado purulento, entre otros indicadores «no directos» de infección.	Alemania
11	2020/Wound Infection in Clinical Practice -Principles of best practice^(9)^	Sistema de clasificación	Infection Management Pathway	Heridas agudas y crónicas de diversas etiologías	Signos y síntomas clínicos comoeritema, edema, exudado purulento, dolor, mal olor, retraso de la cicatrización, aumento del eritema, calor, hipergranulación, granulación friable, agrandamiento de la herida, así como otros signos y síntomas y cambios hemodinámicos.	Alemania
12	2020/Validation of the Harikrishna Peripheral Skin Classification for wound assessment^(22)^	Sistema de clasificación	Harikrishna Peripheral Skin Classification (HPSC)	Heridas agudas y crónicas de diversas etiologías	Clasifica la piel perilesional para la infección, utilizando signos como: inflamación sin infección, inflamación con infección, atípica - células senescentes/cáncer/enfisema subcutáneo.	Malasia
13	2020/Management tools in nursing care for children with pressure injury^(23)^	Formulario de inscripción y un Flujograma	Instrumento de sistematización de los cuidados de enfermería para niños con lesiones por presión y Flujograma de riesgo y prevención de lesiones por presión de la Universidad Federal de Espírito Santo	Lesión por presión	Signos y síntomas clínicos comoedema, aumento de la temperatura, hiperemia y aumento del tejido necrótico.	Brasil
14	2019/Use of a bacterial fluorescence imaging device: wound measurement, bacterial detection and targeted debridement^(24)^	Dispositivo	MolecuLight	Úlceras diabéticas, venosas y arteriales, heridas quirúrgicas y lesiones por presión	Evalúa la infección a través de la carga bacteriana.	Canadá
15	2019/Evaluation of Wound Photography for Remote Postoperative Assessment of Surgical Site Infections^(25)^	Registro fotográfico a través del uso de *smartphones*	No se aplica	ISC (Infección de Sítio Quirúrgico)	Signos de infección notificados por los pacientes como presentes o ausentes y uso de imágenes fotográficas, asociadas o no a la notificación de infección presente o ausente.	EUA
16	2018/Importance of postprocedural Wound, Ischemia, and foot Infection (WIfI) restaging in predicting limb salvage^(26)^	Sistema de clasificación	WIfI	Úlceras de miembros inferiores de diversas etiologías	Signos y síntomas clínicos como:infección local que afecte sólo a la piel y al tejido subcutáneo, infección local con eritema >2 cm o que afecte a estructuras más profundas que la piel y los tejidos subcutáneos, e infección local con signos de síndrome de respuesta inflamatoria sistémica.	EUA
17	2018/Diagnostic value of fluorine-18 deoxyglucose positron emission tomography/computed tomography in deep sternal wound infection^(27)^	Tomografía computarizada por emisión de positrones(PET/CT)	No se aplica	Heridas quirúrgicas	Signos de infección a través de la zona comprometida que se muestra en la imagen.	China
18	2018/Elaboration of an algorithm for wound evaluation and treatment^(28)^	Algoritmo	Algoritmo de evaluación y tratamiento de heridas de la Universidad de Vale do Sapucaí	Heridas agudas y crónicas de diversas etiologías	Signos y síntomas clínicos comocalor, eritema, edema, dolor, exudado purulento, así como cambios hemodinámicos.	Brasil
19	2017/The inter-rater reliability between nurse-assessors clinically assessing infection of chronic wounds using the WUWHS criteria^(29)^	Formulario de inscripción	WUWHS	Heridas crónicas de diversas etiologías	Signos y síntomas clínicos como: dolor, retraso en la cicatrización, edema, tejido de granulación friable, mal olor, decoloración, aumento del exudado, induración, eritema, así como cambios hemodinámicos.	Holanda
20	2017/Wound Infection in Clinical Practice -Principles of best practice^(9)^	Sistema de clasificación	WIRE	Heridas agudas y crónicas de diversas etiologías	Signos y síntomas clínicos como: dolor, tejido necrótico, granulación friable, exposición de órganos subyacentes, retraso en la cicatrización, eritema, calor, edema, olor, exudado, entre otros.	Reino Unido
21	2017/Inter-rater and intra-rater reliability outcomes of a rapid bacteria counting system with pressure ulcer samples^(30)^	Dispositivo Panasonic Healthcare DU-AA01NP-H	No se aplica	Lesión por presión	Evalúa la infección a través de la carga bacteriana.	Japón
22	2016/The Wound Trend Scale: A Retrospective Review of Utility and Predictive Value in the Assessment and Documentation of Lower Leg Ulcers^(31)^	Escala	WTS	Úlceras de miembros inferiores de diversas etiologías	Signos y síntomas clínicos como: exudado, tejido necrótico y desbridamiento, profundidad, purulencia, piel periherida, edema, dolor y riesgo de infección (eritema, calor, edema y dolor, así como detección de infección mediante pruebas).	Canadá
23	2016/Evaluation of a Surgical Site Discharge Teaching Tool Using Pictures and a Mirror^(32)^	Educación sanitaria para enseñar a autoevaluar las incisiones quirúrgicas.	No se aplica	Heridas quirúrgicas, concretamente tras laparotomía	Signos y síntomas clínicos como: calor, supuración, exudado purulento, eritema, edema, dolor/sensibilidad, picor, entre otros signos y síntomas.	EUA
24	2015/Adaptação cultural e validação para a população portuguesa de um instrumento de monitorização de feridas crónicas: escala RESVECH 2.0^(33)^	Escala	RESVECH 2.0	Heridas crónicas de diversas etiologías	Signos y síntomas clínicos como: dolor; eritema, edema, calor, aumento del exudado, exudado purulento, tejido friable, herida estancada, tejido compatible con biofilm, olor, hipergranulación, aumento del tamaño de la herida, lesiones satélites, palidez tisular, entre otros signos y síntomas.	Portugal
25	2015/Construção de instrumento de coleta de dados para pessoas com feridas embasado na teoria de Wanda de Aguiar Horta^(34)^	Formulario de inscripción	Instrumento de recogida de datos para personas con heridas basado en la teoría de Wanda de Aguiar Horta de la Universidad Federal de Paraíba	Úlcera diabética	Sinais e sintomas clínicos como: edema, calor, eritema, aumento de tecido, dor e exsudato.	Brasil
26	2013/Reliability and validity of the Chinese version of DESIGN-R, an assessment instrument for pressure ulcers^(35)^	Formulario de inscripción	DESIGN-R	Lesión por presión	Signos y síntomas clínicos, pero no específicos de cuáles, además de otras características generales evaluadas de la herida como: profundidad de la herida, exudado, tamaño, infección, granulación y necrosis.	China
27	2012/A systematic review of the ASEPSIS scoring system used in non-cardiac-related surgery^(36)^	Sistema de clasificación	ASEPSIS	Heridas quirúrgicas	Signos y síntomas clínicos, pero no específicos, además de otras características generales evaluadas de la herida como: secreción serosa, eritema, exudado purulento, separación profunda del tejido, aislamiento de bacterias y hospitalización durante más de 14 días.	Reino Unido
28	2006/Wound Infection in Clinical Practice -Principles of best practice^(9)^	Sistema de clasificación	NERDS and STONES	Heridas crónicas de diversas etiologías	Signos y síntomas clínicos como: heridas que no cicatrizan, heridas exudativas, tejido de granulación rojo y sangrante en la superficie de la herida, restos en la superficie de la herida, olor u olor desagradable de la herida, aumento del tamaño, aumento de la temperatura, palpación o exposición del hueso, zonas de nueva división o lesiones *satélites*, exudado, eritema, edema.	Canadá
29	2001/A tool to assess clinical signs and symptoms of localized infection in chronic wounds: development and reliability^(37)^	Lista de verificación	Clinical Signs and Symptoms Checklist (CSSC)	Heridas crónicas de diversas etiologías	Signos y síntomas clínicos comoaumento del dolor en la zona de la úlcera, eritema, edema, calor, exudado purulento, exudado seroso asociado a inflamación, retraso en la cicatrización de la úlcera, decoloración del tejido de granulación, tejido de granulación friable, mal olor, heridas satélites y formación de bolsas en la base de la úlcera.	EUA
30	1997/The Sessing Scale for measurement of pressure ulcer healing^(38)^	Escala	Sessing Scale	Lesión por presión	Signos y síntomas clínicos comodrenaje purulento, mal olor, tejido necrótico, así como síntomas sépticos.	EUA
31	1997/Monitoring wound healing by odour^(39)^	Dispositivo con sensores	AromaScan	Úlcera vascular	Aroma de las lesiones y su relación con los microorganismos presentes en la herida.	Reino Unido
32	1995/Wound registry: development and validation^(40)^	Formulario	Formulario de llenado de la Universidad Estadual de Nova York	Heridas traumáticas	Signos y síntomas clínicos como:Eritema, calor, sensibilidad o supuración, combinados con el juicio clínico, así como valorar el aspecto estético en poco tiempo.	EUA

Para ayudar a identificar las principales características de las infecciones en los estudios analizados, se creó una nube de palabras, ilustrada en la Figura 2. Esta representación visual destaca los principales signos y síntomas clínicos de las infecciones de heridas. Los datos presentados reflejan la información más prevalente encontrada en los estudios evaluados.

**Figura 2:** Nube de palabras de signos y síntomas frecuentes en las publicaciones. Río de Janeiro, RJ, Brasil, 2024.

Los resultados de esta revisión identificaron 32 herramientas para evaluar la infección de heridas, con publicaciones que oscilan entre 1995 y 2023, 13 de las cuales se publicaron en los últimos 5 años. La mayoría de las herramientas encontradas evalúan la infección de forma cualitativa, a través de signos y síntomas clínicos, correspondiendo a 26 instrumentos^(9, 14, 15, 16, 17, 18, 20, 21, 22, 23, 26, 28, 29, 31, 32, 33, 34, 35, 36, 37, 38, 40, 41, 42)^. Las demás evalúan utilizando recursos tecnológicos más sofisticados, como equipos y dispositivos electrónicos, totalizando 5 herramientas, y sólo 1 utiliza *software* para evaluar la lesión y la infección^(19, 24, 25, 27, 30, 39)^.

Las herramientas que evalúan la infección a través de los signos y síntomas que presenta la herida son construcciones tradicionales que se presentan de diversas formas, desde formularios, escalas, sistemas de clasificación, *listas de comprobación*, acrónimos, folletos explicativos y algoritmos, y pueden imprimirse en papel para rellenar con bolígrafo o en plataformas digitales. Estas herramientas evalúan la infección mediante la observación directa de la piel, el lecho de la lesión y la piel perilesional. Los signos y síntomas de infección evaluados varían en función de las particularidades de cada constructo y de la etiología de la herida evaluada^(9, 14, 15, 16, 17, 18, 20, 21, 22, 23, 26, 28, 29, 31, 31, 32, 33, 34, 35, 36, 37, 38, 40, 41, 42)^.

Entre las herramientas que evalúan las infecciones de las heridas mediante recursos tecnológicos se encuentran los equipos que utilizan imágenes por emisión de positrones combinadas con tomografía computarizada, la evaluación cuantitativa de la carga microbiana mediante imágenes de inmunofluorescencia, los *programas informáticos* para registrar, monitorizar y analizar las heridas (incluidos los signos y síntomas de infección), los sensores para evaluar el olor como indicador predictivo de infección y el uso de *teléfonos inteligentes* para ayudar a identificar complicaciones de las heridas como las infecciones^(19, 24, 25, 27, 30, 39)^.

Entre las herramientas tradicionales que evalúan la infección basándose en los signos y síntomas clínicos se encuentran la *lista de comprobación de signos y síntomas clínicos* (CSSC), el índice terapéutico para infecciones locales (TILI), *la vía de gestión de infecciones, Wound Infection Continuum – International Wound Infection Institute* (WIC- IWII), *World Union of Wound Healing Societies* (WUWHS) y los acrónimos ASEPSIS, WIRE, NERDS y STONES, WIfI, IWGDF/IDSA *System* ^(9, 21, 26, 29, 36, 37, 41, 42)^. También se identificaron otros instrumentos en los estudios analizados, aunque sin nombres específicos^(14, 15, 16, 17, 18, 23, 28, 32, 34, 40)^. Los signos y síntomas clásicos de infección mencionados en la mayoría de estos instrumentos incluyen dolor, calor, edema, eritema, exudado purulento y olor^(9, 14, 15, 16, 17, 18, 20, 21, 22, 23, 26, 28, 29, 31, 32, 33, 34, 35, 36, 37, 38, 40, 41, 42)^.

Sólo las siglas NERDS y STONES han definido el número de signos y síntomas necesarios para clasificar la infección en una herida^(9)^. Ninguna otra herramienta ha especificado el número de características clínicas que deben estar presentes en la lesión para diagnosticar una infección^(9, 21, 26, 29, 36, 37, 41, 42)^. Como señalan las herramientas tradicionales, ningún signo o síntoma por sí solo determina de forma fiable la presencia o ausencia de infección en una herida. Por lo tanto, debe utilizarse la combinación de múltiples signos y síntomas indicativos de infección para obtener un diagnóstico clínico más preciso y fiable^(9, 29)^.

Los instrumentos también identificaron que algunos signos y síntomas son más específicos y sensibles como predictores de infección que otros. Además, algunos signos pueden manifestarse de forma sutil o incluso no manifestarse en absoluto, dependiendo del tipo de herida y de las condiciones clínicas del paciente^(9, 34, 37, 41)^. Los estudios también han demostrado que los signos de inflamación -calor, eritema, edema y dolor- pueden estar presentes en heridas infectadas y se consideran signos clásicos de infección^(9, 14, 15, 16, 17, 18, 19, 20, 23, 24, 27, 30, 31, 32, 33, 34, 36, 39, 40)^.

Otras herramientas analizadas incluyen un ítem o dominio que evalúa la infección, entre otras características, aunque no fueron diseñadas específicamente para este fin. Entre estas herramientas se encuentran RESVECH 2.0, DESIGN-R, *Harikrishna Periwound Skin Classification* (HPSC), *Wound Trend Scale* (WTS), *Sessing Scale*, el DMIST y SWAT mneumonic, así como otras herramientas sin nombre específico. Los signos y síntomas clínicos de infección más frecuentemente identificados son: exudado purulento, mal olor, eritema, edema^(16, 20, 22, 31, 33, 35, 38)^.

En cuanto a las herramientas que evalúan cuantitativamente la carga microbiana presente en el lecho de la lesión y su concentración, destacan el dispositivo *MolecuLight* y el dispositivo de Panasonic *Healthcare* DU-AA٠١NP-H. Estos dispositivos permiten evaluar la infección a pie de cama de una forma menos invasiva y más rápida^(24, 30)^. Además, el dispositivo AromaScan ayuda a monitorizar el progreso de la herida e identificar una posible infección basándose en el patrón de olor detectado. Los sensores de polímeros conductores, por su parte, muestran cambios en la resistencia eléctrica cuando se exponen a una mezcla de sustancias químicas volátiles^(39)^.

El uso de *teléfonos inteligentes*, que también se encuentra en el mapeo de herramientas de esta investigación, puede utilizarse para ayudar a evaluar las complicaciones en las heridas quirúrgicas, como la ISQ (infección del sitio quirúrgico) o la SSO (ocurrencia del sitio quirúrgico), incluidos el seroma, la celulitis, el hematoma, la dehiscencia y el absceso^(25)^. En cuanto a la tomografía computerizada, esta tecnología ha demostrado ser una posibilidad para identificar la infección en estructuras más profundas de la herida^(27)^.

En cuanto a la distribución geográfica de las herramientas, se identificaron 18 en el continente americano, 7 en Brasil, 4 en Canadá y 7 en Estados Unidos^(9, 14, 15, 17, 18, 19, 23, 24, 25, 26, 28, 31, 32, 34, 37, 38, 40)^. En Asia, se localizaron 6 herramientas: 1 en Vietnam, 2 en Japón, 2 en China y 1 en Malasia^(16, 20, 22, 30, 35)^. En Europa, se encontraron 8 herramientas, repartidas entre Alemania (2), Países Bajos (2), Reino Unido (3) y Portugal (1)^(9, 21, 29, 33, 36)^. En los demás continentes, no se identificaron trabajos publicados en esta revisión exploratoria.

De las diez herramientas clínicas tradicionales específicas para evaluar la infección a través de signos y síntomas, seis proceden de Europa^(9, 21, 29, 36)^ y cuatro de América^(9, 26, 37)^. Los demás presentan algún ítem o dominio para evaluar la infección, pero no son específicos para esta finalidad^(14, 15, 16, 17, 18, 19, 20, 22, 23, 28, 31, 32, 33, 34, 35, 38, 40)^. Ninguno de los estudios brasileños mapeados en esta revisión evalúa exclusivamente el proceso infeccioso en heridas^(14, 17, 18, 19, 23, 28, 34)^.

## DISCUSIÓN

El mapeo de la evidencia disponible en la literatura revela que las herramientas de evaluación de la infección de heridas tienen diferente aplicabilidad, con predominio de instrumentos basados en enfoques tradicionales, que evalúan la infección a través de los signos y síntomas clínicos observados en las lesiones. Este hallazgo coincide con las principales directrices internacionales, que recomiendan analizar las características de la herida para diagnosticar la infección. Este enfoque destaca por ser práctico, accesible, rápido y factible de aplicar en diferentes escenarios clínicos, además de tener una buena validez y fiabilidad, lo que permite un diagnóstico precoz^(9, 14, 15, 16, 17, 18, 20, 21, 22, 23, 26, 28, 29, 31, 32, 33, 34, 35, 36, 37, 38, 40, 41, 42)^.

Los signos clínicos desempeñan un papel fundamental en el seguimiento de la evolución de las heridas infectadas y son elementos clave para orientar las decisiones terapéuticas y evaluar la respuesta al tratamiento^(8, 10, 29, 31, 33)^. Las herramientas de evaluación permiten organizar estos signos de forma sistemática, favoreciendo un seguimiento estructurado y más eficiente^(7, 9, 11)^. El análisis cuidadoso de los signos clínicos ayuda a los profesionales sanitarios a identificar cambios tempranos en el estado de la lesión, lo que permite ajustar a tiempo las intervenciones terapéuticas y contribuye a mejorar los resultados clínicos^(8, 10)^.

La mayoría de las herramientas que evalúan la infección basándose en los signos y síntomas clínicos incluyen los signos cardinales de la inflamación: dolor, calor, edema y eritema. Esto se justifica por el hecho de que el proceso inflamatorio está presente en las heridas infectadas, lo que hace que estos signos también sean indicadores relevantes de infección. Se sabe que no son específicos de infección si se evalúan de forma aislada, pero deben considerarse predictivos de infección en heridas que muestren estas características sin una causa identificada. Según algunas herramientas, los signos y síntomas de infección se siguen clasificando en signos clásicos, sutiles y sistémicos^(8, 9, 14, 20, 21, 26, 29, 41, 42)^.

Las herramientas tecnológicas para evaluar la infección de heridas son menos frecuentes en la bibliografía, posiblemente debido a su elevado coste y a su limitada disponibilidad^(24, 27, 30, 39)^. Sin embargo, estas tecnologías pueden ser especialmente útiles en contextos que requieren mayor precisión y agilidad. Los estudios revisados sugieren que se necesita más investigación para consolidar el uso de estas tecnologías en la práctica clínica. A pesar de ello, ya han demostrado potencial para ofrecer un diagnóstico rápido, seguro y menos invasivo, favoreciendo un tratamiento a tiempo y una gestión eficaz de la infección^(9, 19, 24, 25, 27, 30, 39)^.

En cuanto a los tipos de heridas evaluados, existe diversidad de etiologías, abarcando tanto heridas agudas como crónicas, lo que posibilita su aplicación en diferentes contextos asistenciales, como atención primaria, domiciliaria y hospitalaria^(9, 21, 26, 29, 36, 37, 41, 42)^. Este hallazgo es similar a los resultados de Cardinelli et al.^(11)^, que también identificaron herramientas de evaluación aplicables a diferentes etiologías y escenarios clínicos.

La literatura presenta un amplio abanico de instrumentos para la evaluación de heridas infectadas, desde herramientas completas capaces de evaluar múltiples lesiones hasta otras más específicas para determinadas etiologías^(9, 14, 20, 26, 28, 29, 31, 40)^. El conocimiento de estas herramientas y de sus particularidades es esencial para que los profesionales sanitarios puedan adaptar los cuidados de forma más precisa, segura y eficaz(^11^).

El uso de instrumentos de evaluación de heridas ha demostrado ser una tecnología capaz de mejorar la calidad de la atención al paciente, ya que orienta la práctica clínica en el tratamiento y manejo de estas lesiones basándose en la mejor evidencia, y su uso permite estandarizar el análisis de las heridas, reduciendo la subjetividad en la evaluación. Aunque la literatura apunta a estas herramientas como una posibilidad para mejorar la calidad asistencial, existen pocos estudios publicados que demuestren la efectividad de su uso rutinario y sistematizado en la práctica clínica^(11, 43)^.

La aplicación de estas tecnologías en el manejo de pacientes con heridas puede tener beneficios significativos, por lo que es necesaria una evaluación exhaustiva para realizar la elección correcta, dependiendo del contexto en el que se manejen las heridas y del objetivo de la evaluación. Además, es esencial comprender cómo utilizar correctamente estas herramientas para evitar su uso inadecuado y, en consecuencia, evaluaciones erróneas^(11, 43)^.

En cuanto a la evaluación de infecciones, el consenso *del Instituto Internacional de Infecciones de Heridas* (IWII, *International Wound Infection Institute* por la sigla en inglés), formulado en 2022, presenta herramientas específicas para identificar infecciones de heridas de diversas etiologías. Algunas de ellas se encuentran en fase de validación psicométrica, mientras que otras ya han sido validadas para su uso clínico. También incluyen un análisis de los factores de riesgo de infección(^9^).

En el contexto brasileño, una revisión de alcance mapeó 51 instrumentos disponibles en la literatura para la evaluación de heridas, de los cuales sólo ocho han sido adaptados y validados para su uso en Brasil. Sin embargo, ninguna de estas herramientas es específica para las infecciones de heridas. La mayoría de las herramientas traducidas al portugués se centran en la evaluación del proceso de cicatrización, la clasificación de las lesiones y la preparación adecuada del lecho de la herida para optimizar el tratamiento^(11)^.

Además de la variedad de instrumentos disponibles, es crucial tener en cuenta la validez y fiabilidad de las herramientas de evaluación. No existe una herramienta «patrón oro» para identificar las infecciones de las heridas, ya que esta elección depende del tipo de lesión, del escenario de evaluación y de los recursos disponibles. Además, el equipo sanitario debe estar debidamente formado y cualificado para utilizar las herramientas de evaluación de infecciones de forma segura y eficaz. Independientemente del instrumento elegido, se sabe que una herramienta fiable y validada ayuda en el cuidado de pacientes con infecciones de heridas, previniendo complicaciones y promoviendo una mejor toma de decisiones^(9, 11, 43)^.

A pesar de los avances de la ciencia y la tecnología aplicadas al cuidado de heridas, sigue habiendo una laguna importante en la sistematización de la evaluación y validación de instrumentos específicos para identificar infecciones de heridas. Esta revisión exhaustiva pretende contribuir a colmar esta laguna mediante el mapeo de las herramientas existentes, proporcionando una visión global de sus características. Además, la investigación pone de relieve la falta de instrumentos validados en el contexto brasileño para evaluar la infección.

Los resultados de esta revisión proporcionan una base para futuras investigaciones clínicas y estudios metodológicos destinados a crear y validar herramientas, fortaleciendo la práctica basada en la evidencia. Al sistematizar los datos, esta revisión pretende ayudar a los profesionales sanitarios a elegir métodos adecuados para diagnosticar y tratar las infecciones de heridas, promoviendo una atención estandarizada y, en consecuencia, mejores resultados clínicos para los pacientes.

## CONCLUSIÓN

La diversidad de herramientas de evaluación de infecciones de heridas disponibles en la bibliografía refleja la complejidad y multidimensionalidad del tratamiento de las heridas infectadas. Estas herramientas abarcan desde sencillos formularios de cumplimentación manual y valoración global de la lesión, que cubren diferentes aspectos como la presencia de signos y síntomas clínicos de infección, hasta el uso de equipos más avanzados y tecnológicos para el análisis de la carga microbiana y pruebas de imagen.

Las herramientas de evaluación más utilizadas en la práctica clínica se basan en los signos clínicos, y en Brasil no existen instrumentos validados para identificar específicamente las infecciones de las heridas. Este estudio sugiere el desarrollo de herramientas adaptadas a la realidad brasileña y la realización de nuevas investigaciones para validar la fiabilidad, los beneficios y las limitaciones de estos instrumentos en la práctica asistencial.
